# Methane and nitrous oxide budget for Chinese natural terrestrial ecosystems

**DOI:** 10.1093/nsr/nwaf094

**Published:** 2025-03-11

**Authors:** Tingting Li, Xinyi Liu, Jiahui Tian, Wenping Yuan, Xuhui Wang, Xiu-Qun Yang, Songbai Hong, Yilong Wang, Qiuan Zhu, Lijun Yu, Jiangzhou Xia, Han Xiao, Minqi Liang, Shihua Li, Zimeng Li, Yingxuan Wang, Kerou Zhang, Min Xu, Zhangcai Qin

**Affiliations:** State Key Laboratory of Atmospheric Environment and Extreme Meteorology, Institute of Atmospheric Physics, Chinese Academy of Sciences, Beijing 100029, China; International Research Center of Big Data for Sustainable Development Goals, Beijing 100094, China; State Key Laboratory of Atmospheric Environment and Extreme Meteorology, Institute of Atmospheric Physics, Chinese Academy of Sciences, Beijing 100029, China; College of Earth and Planetary Sciences, University of Chinese Academy of Sciences, Beijing 101408, China; State Key Laboratory of Atmospheric Environment and Extreme Meteorology, Institute of Atmospheric Physics, Chinese Academy of Sciences, Beijing 100029, China; College of Earth and Planetary Sciences, University of Chinese Academy of Sciences, Beijing 101408, China; International Research Center of Big Data for Sustainable Development Goals, Beijing 100094, China; Institute of Carbon Neutrality, Sino-French Institute for Earth System Science, College of Urban and Environmental Sciences, Peking University, Beijing 100871, China; Institute of Carbon Neutrality, Sino-French Institute for Earth System Science, College of Urban and Environmental Sciences, Peking University, Beijing 100871, China; School of Atmospheric Sciences, Nanjing University, Nanjing 210023, China; School of Urban Planning and Design, Shenzhen Graduate School, Peking University, Shenzhen 518055, China; State Key Laboratory of Tibetan Plateau Earth System, Resources and Environment (TPESRE), Institute of Tibetan Plateau Research, Chinese Academy of Sciences, Beijing 100101, China; College of Geography and Remote Sensing, Hohai University, Nanjing 211100, China; Aerospace Information Research Institute, Henan Academy of Sciences, Zhengzhou 450046, China; Tianjin Key Laboratory of Water Resources and Environment, Tianjin Normal University, Tianjin 300387, China; School of Atmospheric Sciences, Guangdong Province Data Center of Terrestrial and Marine Ecosystems Carbon Cycle, Sun Yat-sen University, Zhuhai 519000, China; School of Atmospheric Sciences, Guangdong Province Data Center of Terrestrial and Marine Ecosystems Carbon Cycle, Sun Yat-sen University, Zhuhai 519000, China; School of Atmospheric Sciences, Guangdong Province Data Center of Terrestrial and Marine Ecosystems Carbon Cycle, Sun Yat-sen University, Zhuhai 519000, China; Institute of Carbon Neutrality, Sino-French Institute for Earth System Science, College of Urban and Environmental Sciences, Peking University, Beijing 100871, China; Institute of Carbon Neutrality, Sino-French Institute for Earth System Science, College of Urban and Environmental Sciences, Peking University, Beijing 100871, China; Wetland Research Center, Institute of Ecological Conservation and Restoration, Chinese Academy of Forestry, Beijing 100091, China; College of Geography and Remote Sensing, Hohai University, Nanjing 211100, China; International Research Center of Big Data for Sustainable Development Goals, Beijing 100094, China; School of Atmospheric Sciences, Guangdong Province Data Center of Terrestrial and Marine Ecosystems Carbon Cycle, Sun Yat-sen University, Zhuhai 519000, China

**Keywords:** methane, nitrous oxide, China, natural terrestrial ecosystems, budget

## Abstract

China's natural terrestrial ecosystems (NTEs) are significant sources and sinks of methane (CH₄) and nitrous oxide (N₂O), two potent non-CO₂ greenhouse gases. This article reviews CH₄ and N₂O inventories for China's NTEs, derived from site-specific extrapolation and models, to elucidate their spatiotemporal emission patterns. Despite progress, significant gaps remain, including large uncertainties due to model limitations and inconsistent driving data, insufficient assessments of integrated global warming potential (GWP) under long-term land-use and climate changes, the lack of freshwater emission inventories, and the need for more observations, refined prior sectoral contributions, and novel methods like isotopic signature applications in machine-learning and inversion techniques. This review offers a new perspective by compiling a new CH₄ and N₂O inventory and evaluating their integrated GWP for 1980–2020, developed using multi-model approaches to assess climate and land-use impacts. The review underscores the importance of CH₄ and N₂O sources and sinks, offering recommendations to enhance carbon sequestration and reduce emissions.

## Teaser

China’s natural ecosystems play a crucial role in the balance of methane and nitrous oxide, two powerful greenhouse gases. This review highlights their emission patterns, gaps in current understanding, and offers strategies to better manage emissions and enhance carbon sequestration.

## INTRODUCTION

The globally averaged surface mole fractions of methane (CH_4_) and nitrous oxide (N_2_O) reached new highs in 2021, with CH_4_ at 1908 ± 2 ppb and N_2_O at 334.5 ± 0.1 ppb, which constitute 262% and 124% of pre-industrial levels, respectively [1]. Although the CH_4_ and N_2_O concentration is much lower than that of ambient carbon dioxide, both of them highly affect the climate system with radiative efficiencies that are 27 and 273 times greater than CO_2_ at a 100-year time horizon [2]. Around 90% of nationally determined contributions (NDCs) cover targets for CH_4_ and N_2_O [3]. However, compared with intensive studies on terrestrial CO_2_ budget, much less work has been done on quantifying the magnitude and presenting the spatiotemporal patterns of CH_4_ and N_2_O fluxes in natural terrestrial ecosystems (NTEs) [4].

The NTEs are important sources and sinks of the global CH_4_ and N_2_O budgets. The natural wetlands are the largest natural CH_4_ source, contributing ∼60% of the total natural emissions [5]. Upland soils, dominated by forests, grasslands and shrublands, are the main biotic sink for atmospheric CH_4_ [5]. For global N_2_O inventories, the sources from NTEs account for one-third of the total emissions [6]. The CH_4_ and N_2_O exchanges from NTEs present distinct spatiotemporal variations, since their cycling processes are regulated by the climate, edaphic and other biophysical drivers [7,8]. For example, temperature [9] and water table depth [10] could affect archaeal CH_4_ production from wetlands. The CH_4_ oxidation rate of upland soils depends primarily on soil conditions including texture, bulk density, soil organic carbon, moisture and temperature [11]. Soil moisture, soil temperature and atmospheric nitrogen (N) deposition are important drivers for N_2_O emission in NTEs [7,12].

Over the past decades, efforts have been made to estimate regional and global CH_4_ and N_2_O budgets of NTEs [5,6,13–17]. Compared with the previous method of extrapolating field measurements to a given area, recent methodologies, such as process-based models, machine learning (ML) and the satellite-based inversion method, have been improved and become popular [18,19]. These superior methodologies could reduce the uncertainty in estimating greenhouse gas (GHG) budget. The process-based models consider the primary processes and mechanisms [19], the ML technology has the advantage of considering non-linear and interactive relationships among predictors [20], and satellite observations offer important ‘top-down’ information for evaluating ‘bottom-up’ inventories. However, the present inventories still cannot provide a full picture of CH_4_ and N_2_O emissions from NTEs, due to poor reproducing regional observations by models and inversions [19] and missing important data such as land management, flux observations, tall tower observations [4] and satellite observations [18] in key regions.

Based on the third national land survey, China has 284 M ha of forest, 265 M ha of grassland and 23 M ha of wetland, which are vital CH_4_ and N_2_O sources and sinks [13,17,21,22]. Over the past four decades, the NTEs in China underwent dramatic variations due to human activities, such as forest expansion [23], grassland degradation [24] and wetland conversion [25], which would inevitably influence CH_4_ and N_2_O budgets. The National Greenhouse Gas Inventories (NGHGIs) released by the Chinese government do not estimate CH_4_ uptake and N_2_O emissions from NTEs [26,27]. In terms of the Chinese national CH_4_ and N_2_O budgets from NTEs, lots of previous studies evaluated a single sector budget [13,15–17,21], but a synthetic review and a comprehensive long-term evaluation of CH_4_ and N_2_O are deficient.

This review begins with a brief summary of the key processes and controlling factors of CH₄ and N₂O dynamics, along with their evaluation methodologies, to provide background context. It then examines the current national CH₄ and N₂O inventories from China's NTEs. Furthermore, this study identifies the limitations of existing inventories, introduces comprehensive CH₄ and N₂O inventories spanning four decades, and evaluates their overall greenhouse effect from China's NTEs.

## MAJOR PROCESSES AND CONTROLLING FACTORS

In this section, we make a brief summary of the chemical formulas, major biological processes and controlling factors of CH_4_ and N_2_O dynamics from NTEs, as this topic has been well identified by several reviews [7,8,28].

### Methane

**Figure 1.**
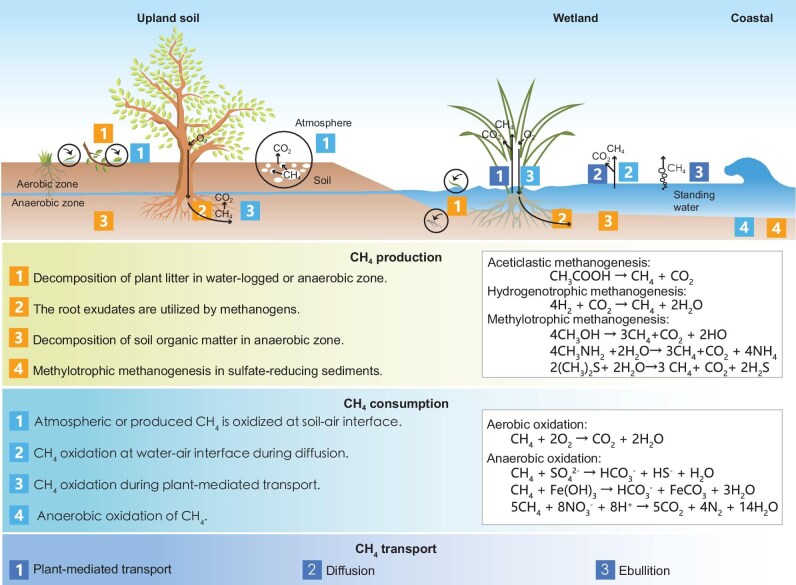
Major processes and chemical reactions involved in CH_4_ dynamics across upland soils, inland and coastal wetlands. CH₄ production pathways include decomposition of plant litter and soil organic matter, utilization of root exudates in anaerobic conditions, and methylotrophic methanogenesis in sulfate-reducing sediments. CH₄ consumption occurs through oxidation at soil–air and water–air interfaces, during plant-mediated transport and in anaerobic zones. CH₄ transport involves plant-mediated pathways, diffusion and ebullition. Key chemical reactions for methanogenesis and oxidation are also illustrated.

CH_4_ is the final product of the anaerobic decomposition of organic matter via methanogens and is oxidized into CO_2_ by methanotrophs. Methanogenesis is a terminal process in anaerobic biomass degradation, commonly seen in the habitats where terminal electron acceptors, such as oxygen, nitrate, iron (III) and sulfate, are missing or rapidly depleted. The complex organic polymers are firstly decomposed to alcohols, short chain fatty acids, CO_2_ and H_2_ via hydrolytic and fermenting bacteria. Then, microbiological methanogenesis appears through three pathways: acetoclastic, hydrogenotrophic and methylotrophic methanogenesis (Fig. 1) [28]. Aerobic and anaerobic oxidations are two important pathways of microbial CH_4_ oxidation [29]. Further details on methanogenesis and CH₄ oxidation pathways are given in Fig. 1 and Supplementary S1.1.

Among NTEs, wetlands are the largest natural source of CH_4_ globally, while upland forests and grasslands on freely drained soils are deemed to be CH_4_ sinks [5]. CH_4_ production, oxidation and transport are the main processes that determine the CH_4_ fluxes in NTEs (Fig. 1). The root exudates and organic matter, i.e. plant litter and soil organic matter, could supply substrates for methanogenesis in the anaerobic environment [19]. Vascular plant and tree stem, ebullition, and diffusion are three plant-mediated pathways of CH_4_ transport. In natural wetlands, plant-mediated CH_4_ transport represents the dominant pathway. However, the efficiency and characteristics of this transport mechanism vary significantly among different plant species and under diverse environmental conditions [30]. CH_4_ oxidation occurs at the soil–atmosphere or water–atmosphere interface, and around roots where O_2_ diffuses into anaerobic soil via plant transport [31]. Soil conditions, such as soil temperature, moisture, pH, bulk density, texture, nutrients and organic carbon content, could regulate the CH_4_ fluxes (Supplementary S1.2).

### Nitrous oxide

**Figure 2.**
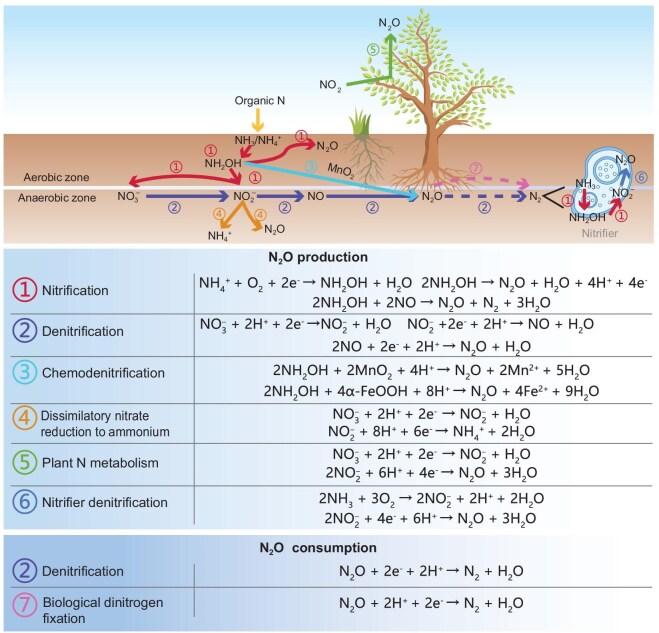
Major processes and chemical reactions involved in N₂O dynamics within natural terrestrial ecosystems. N₂O production pathways include (1) nitrification, (2) denitrification, (3) chemodenitrification, (4) dissimilatory nitrate reduction to ammonium (DNRA), (5) plant nitrogen metabolism and (6) nitrifier denitrification. N₂O consumption occurs through (2) denitrification and (7) biological dinitrogen fixation. The diagram also illustrates key chemical reactions for N_2_O production and consumption in aerobic and anaerobic zones.

In NTEs, N_2_O is a product of such processes as nitrification, denitrification, nitrifier denitrification, chemodenitrification, dissimilatory nitrate reduction to ammonium (DNRA), and plant nitrogen metabolism [7] (Fig. 2). Denitrification and nitrification are the main processes producing N_2_O, which may contribute 70% of the total N_2_O emissions [7]. Further details on the pathways of such processes are described in Supplementary S1.3. The major N_2_O consumption occurs during the last step of denitrification, which is reducing N_2_O to N_2_ [7]. In addition, biological dinitrogen fixation (BNF) could also reduce N_2_O to N_2_ or NH_3_ [7]. The produced N_2_O in the soil could be transported with groundwater whenever it reaches the surface [32]. It could also be transported by plant aerenchyma [33].

Both biological and non-biological factors have an influence on N_2_O emission. Microorganisms and plants are two important biological factors. The abundance, type, structure, physiology and biogeographical patterns of microorganisms have predominant effects on N_2_O emission. For example, the soil crenarchaeotic group shows a strong positive correlation with N_2_O emission [34]. However, environmental factors may be the dominant determinants of its activity. Plants could affect N_2_O production and transport through plant leaf nitrate assimilation and by providing conduits [33], respectively. In addition to biological controls, there are some non-biological controls of N_2_O production and consumption, such as soil moisture, soil temperature, soil pH and N deposition (Supplementary S1.4).

## METHODOLOGIES FOR CH_4_ AND N_2_O BUDGET ESTIMATION

The data-driven method, process-based models and atmospheric inversion method are commonly used to estimate CH_4_ and N_2_O budgets from NTEs. The first two methods belong to bottom-up approaches, which support the United Nations Framework Convention on Climate Change and the Copenhagen Accord. The atmospheric inversion method, as a top-down approach, is also very important in the context of verification of international agreements on emission reductions in recent decades. This section briefly reviews the above three methods in estimating CH_4_ and N_2_O budgets from NTEs.

### Data-driven method

The data-driven method is based on site-specific CH_4_ and N_2_O flux observations. As more observations have been conducted, this method has been developed and could be divided into three categories: empirical extrapolation, statistical models and ML technique (Fig. S1a). The empirical extrapolation method extrapolates the field flux measurements into a given area to get the regional GHG emissions, while the statistical models incorporate the empirical relationship between the fluxes and multiple environmental factors based on abundant field observations (Supplementary S2.1). They are simple data-driven methods compared to the ML technique.

With increasing observations, the ML approach has been increasingly applied to generate new CH₄ and N₂O inventories. Algorithms such as support vector machine (SVM), random forest (RF) and extreme gradient boosting have been used to predict GHG emissions from NTEs [35]. The ML method is effective for generating inventories in small areas with sufficient data but is rarely applied to large-scale regional CH₄ and N₂O budgets (Supplementary S2.1). In addition, as the ML method relies on historical observational data, it cannot account for future changes and is therefore currently unsuitable for future predictions.

### Process-based models

Process-based models, used for understanding carbon and nitrogen cycling in response to environment and quantifying budgets, are the highest intergovernmental panel on climate change (IPCC)-recommended tier (Tier 3) for national inventories. Over the past decades, CH₄ and N₂O models have become increasingly comprehensive and mechanistic (Fig. S1b) and are now the most widely used method for estimating CH₄ and N₂O inventories at both small and large scales. Earlier models, like Christensen's [36] and CASA [37], treated CH₄ flux as a proportion of productivity or respiration. Later models (Table S1) introduced complex frameworks to simulate methanogenesis using different methanogenic substrates, such as soil organic matter, and dissolved organic carbon, methanotrophy by dual Monod Michaelis–Menten-like equations or diffusion-reaction equations, and at least one transport pathway (plant-mediated transport, ebullition and diffusion) (Table S1). Recent advancements, such as the IBIS CH₄ module [38], incorporate microbial interactions. Since 2010, models like SDGVM, CLM and ORCHIDEE_WET (Table S1) have been coupled with Earth System Models to study CH₄ climate feedbacks, though fully coupled simulations remain rare [39,40].

Compared with wetland CH_4_ emissions, fewer models focus on soil CH_4_ uptakes (Table S2). Early models, such as Potter's model [41] and Ridgwell's model [42], used oversimplified diffusion-reaction equations influenced by environmental factors, while later models, like Curry's model [43] and Memo v1.0 [44] refined these equations by including CH_4_ oxidation boundaries (Table S2). After 2011, some CH_4_ emission models incorporated upland CH_4_ oxidation modules, using either diffusion-reaction functions (e.g. LPJ-WHyMe [45] and VISIT [46]) or Michaelis–Menten kinetics (e.g. DLEM [22], TRIPLEX-GHG [16], TEM [47] and CLM4Me [39]).

Earlier models like DAISY, CRISP, CASA and DAYCENT (Table S3) used simplified empirical equations to simulate N₂O production, lacking detailed microbial process descriptions [48]. After 2000, more complex models emerged, such as the DNDC model, which simulates nitrification and denitrification using an ‘anaerobic balloon’ kinetic scheme [49]. The DLEM model incorporates processes like nitrification, denitrification and nitrogen uptake but excludes heterotrophic nitrification and nitrifier denitrification [22]. The IBIS-MicN model accounts for four N₂O production pathways—autotrophic and heterotrophic nitrification, nitrifier denitrification and denitrifier denitrification—while incorporating key microbial mechanisms [50].

### Atmospheric inversion models

Atmospheric inversion of CH_4_ and N_2_O fluxes assimilates column-averaged dry-air mole fractions measurements (e.g. from surface stations, towers, aircraft and satellites) by an inversion system (Table S4). Originating in the 1980s, its development has advanced alongside the development of satellite and tall tower observations [51–53] (Fig. S1c, Supplementary S2.2). Regional and global problems are often ill-conditioned due to limited observations, leading most inversions to rely on Bayesian statistics that integrate observations with prior flux information from inventories or models. Compared to bottom-up approaches, inversion modeling provides accurate global net flux estimates consistent with atmospheric growth rates, avoiding double-counting issues seen in bottom-up methods [5]. However, inversion uncertainties increase at smaller scales, especially in regions with sparse observations, such as the tropics and developing countries [54–56].

## CH_4_ AND N_2_O EMISSIONS FROM CHINA’S NTEs AND THEIR GLOBAL IMPLICATIONS

### A review of CH_4_ and N_2_O budgets from China's NTEs

**Figure 3.**
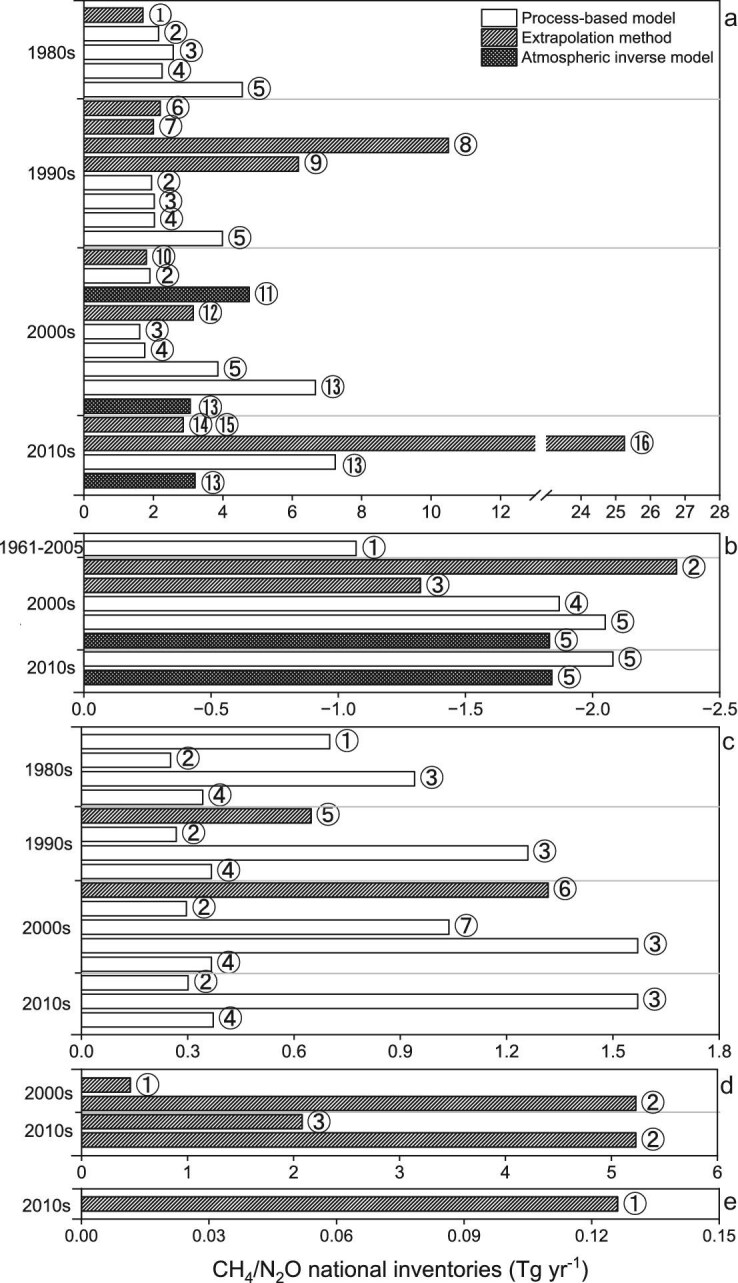
National inventories of (a) CH_4_ emissions from wetlands: (1) Khalil *et al.* (1993) [67], (2) Xu and Tian (2012) [15], (3) Li *et al.* (2015) [13], (4) Zhu *et al.* (2016) [16], (5) Wei and Wang (2016) [14], (6) Wang *et al.* (1993) [68], (7) Jin *et al.* (1999) [69], (8) Wang *et al.* (2012) [70], (9) Cai (2012) [62], (10) Ding and Cai (2007) [71], (11) Zhang *et al.* (2013) [61], (12) Chen *et al.* (2013) [65], (13) Saunois *et al.* (2024) [57], (14) National Greenhouse Gas Inventories (2018) [26], (15) National Greenhouse Gas Inventories (2023) [27] and (16) Xiao *et al.* (2019) [59]; (b) CH_4_ uptake from NTEs excluding freshwaters: (1) Tian *et al.* (2011) [22], (2) Cai (2012) [62], (3) Wang *et al.* (2014) [72], (4) Wei and Wang (2016) [14] and (5) Saunois *et al.* (2024) [57]; (c) N_2_O emissions from NTEs excluding freshwaters: (1) Tian *et al.* (2011) [22], (2) Luo (2015) [64], (3) Tian *et al.* (2019) [58], (4) Liang *et al.* (2024) [17], (5) Chen *et al.* (2000) [63], (6) Cai (2012) [62] and (7) Xu *et al.* (2019) [21]; (d) CH_4_ emissions from freshwaters: (1) Chen *et al.* (2013) [65], (2) Saunois *et al.* (2024) [57], (3) Li *et al.* (2018) [66]; (e) N_2_O emissions from freshwaters: (1) Li *et al.* (2018) [66].

A thorough literature review was conducted, focusing on recent peer-reviewed papers from the Web of Science (for English-language papers; http://apps.webofknowledge.com/) and the China National Knowledge Infrastructure (for Chinese-language papers; https://www.cnki.net/). The literature search utilized the following keywords: CH_4_ and N_2_O emission/uptake/sink/source, wetland, forest, grassland, shrubs, lakes and rivers, freshwater systems, NTEs, upland soils and China. The literature search yielded 15 national inventories for CH_4_ emissions from natural wetlands (Fig. 3a), 4 inventories detailing CH_4_ uptake from upland ecosystem soils (Fig. 3b), 6 inventories on N_2_O emissions from natural soils (Fig. 3c) and only 2 related to lakes (including reservoirs) (Fig. 3d and e). This study also extracted data on N_2_O emissions and CH_4_ uptake from natural soils, as well as CH_4_ emissions from natural wetlands and freshwater systems in Chinese regions, sourced from the data sets released by Global Methane Budget (GMB) [57] and Global Nitrous Oxide Budget (GNB) [4,58], and incorporated into the Chinses national inventories (Fig. 3). Detailed data are provided in Table S5.

The national inventories of wetland CH_4_ emissions are derived by empirical extrapolation methods (10 studies), process-based models (5 studies) and 2 atmospheric inversion studies. The inventories span the period from the 1980s to the 2010s and estimate a range of 1.7 Tg CH_4_ yr^−1^ to 25.3 Tg CH_4_ yr^−1^. This broad range results from the extrapolation methods, which rely on varying flux observations and wetland areas (Fig. 3a and Table S5). The studies before 2010 usually made extrapolations based on fewer flux observations, which may bring large uncertainties. Although the Xiao *et al.* [59] extrapolation was based on 193 studies, there might be an overestimation because of an overestimation of the CH_4_ flux rates from the wetlands on the Qinghai Tibet Plateau [60]. Four process-based models have been used to estimate the Chinese national wetland CH_4_ emissions, including CH4MOD_wetland_ [31], LPJ-WHyMe [14], TRIPLEX-GHG [16] and TEM [15,22]. The simulations cover a long period of 1950‒2010 and show more similar results (Fig. 3a). Between the 1980s and the 2000s, TEM, as well as CH4MOD_wetland_ and TRIPLEX-GHG, obtained relatively low CH_4_ emissions (1.8‒3.3 Tg CH_4_ yr^−1^), with a wetland area of ∼100 000 km^2^ around 2000 (Table S5). However, LPJ-WHyMe presented a higher CH_4_ emission (3.9‒4.6 Tg CH_4_ yr^−1^) based on five wetland area data sets, which is higher than that used in other studies. Only Zhang [61] used the atmospheric inversion method based on atmospheric CH_4_ concentration from SCIAMACHY/ENVISAT to estimate wetland CH_4_ emissions (4.76 Tg CH_4_ yr^−1^). The GMB calculated wetland CH_4_ emissions during the period of 2000‒2019 using both process-based model ensembles and the atmospheric inversion method. The emissions by model ensembles are 6.7‒7.2 Tg CH_4_ yr^−1^, much higher than the above inventories by individual process-based models. The atmospheric inversion models calculated 3.1‒3.2 Tg CH_4_ yr^−1^, which is lower than Zhang's study.

There are five peer-reviewed papers involving the estimation of national CH_4_ uptakes from NTEs, with two papers using extrapolation, two using process-based models and one using both the process-based model and atmospheric inversion method (Fig. 3b). National CH_4_ uptakes are between −2.3 Tg CH_4_ yr^−1^ and −1.1 Tg CH_4_ yr^−1^. No studies gave a long-term trend of the CH_4_ uptakes from NTEs. Although Tian *et al.* [22] simulated CH_4_ uptakes from NTEs between 1961 and 2005, their study only gave an average estimation of −1.1 Tg CH_4_ yr^−1^ during this period. CH_4_ uptakes released by the GMB report, based on both process-based model ensembles and atmospheric inversion models, released a consistent budget range from −2.1 to −1.8 Tg CH_4_ yr^−1^ during the 2000s, with the above studies that focused on China.

The N_2_O emissions from NTEs ranged from 0.25 to 1.57 Tg N_2_O yr^−1^ over the past four decades (Fig. 3c and Table S5). Among the studies, Cai [62] and Chen [63] used the extrapolation method based on observations from 15 forest sites and 6 grassland sites, and they found that the N_2_O emissions are 0.65 Tg N_2_O yr^−1^ and 1.32 Tg N_2_O yr^−1^, respectively. Four process-based models, namely DyN-LPJ [21], DLEM [22], TRIPLEXE-GHG [64] and IBIS-MicN [17], are used to estimate the national N_2_O emissions from NTEs. Although these models have been validated against site-specific N_2_O flux observations, there are some differences in the estimated total national N_2_O emissions. The simulated national N_2_O emissions from NTEs are respectively 0.70 Tg N_2_O yr^−1^ and 1.04 Tg N_2_O yr^−1^ using DLEM and DyN-LPJ during the period of 1961‒2005 and the 2000s. TRIPLEXE-GHG and IBIS-MicN simulated lower and similar values of N_2_O emissions (∼0.3 Tg N_2_O yr^−1^) since they focused on forests and grasslands. Moreover, they both simulated an increasing trend of N_2_O emissions over the past four decades. The GNB also calculated N_2_O emissions from natural soils based on process-based model ensembles, and reported a budget range from 0.9 to 1.6 Tg N_2_O yr^−1^ during the period of 1980‒2016.

There are fewer studies that focus on CH_4_ and N_2_O emissions from freshwaters (Fig. 3d and e), with only two publications focusing on China. All of the budgets are based on the extrapolation method. The GMB calculated 5.23 Tg CH_4_ yr^−1^ during the 2000s and 2010s from freshwaters including lakes, rivers and reservoirs. Publications by Chen [65] and Li [66] both focus on Chinese lakes and reservoirs, and calculated CH_4_ emissions with large differences, in the range of 0.5‒2.1 Tg CH_4_ yr^−1^. Based on Li's calculations, the N_2_O emissions are ∼15% of the CH_4_ emissions from lakes and reservoirs, if converting their 100-year GWP.

### Chinese NTEs’ CH_4_ and N_2_O emission on global budgets

 Since the late 1990s, the Global Carbon Project has periodically released the GMB and GNB reports, detailing CH_4_ and N_2_O budgets from different anthropogenic and natural sectors. It is indicated that global CH_4_ emissions from natural wetlands amount to 162 (131‒214) Tg CH_4_ yr^−1^, while uptakes from upland soils total 33 (11‒49) Tg CH_4_ yr^−1^ during the 2010s [57]. The global N_2_O emission from natural soils is 5.6 (4.9‒6.5) Tg N yr^−1^ between 2007 and 2016 [4]. China's NTEs play a crucial role on a global scale. For example, its forest area ranks fifth in size, its grassland area ranks second, and its wetland area ranks fourth worldwide. This highlights the country's significant contribution to the global CH_4_ and N_2_O budget.

**Figure 4.**
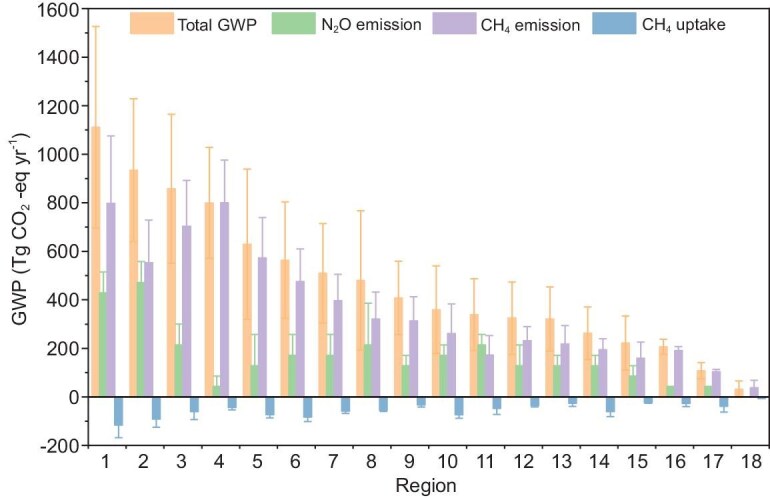
The Global Warming Potential arising from N_2_O and CH_4_ emissions across regions during the 2000s. The N_2_O emissions and CH_4_ uptakes are from natural soils. The CH_4_ emissions are from natural wetlands and freshwaters. Regions: 1 Brazil, 2 Equatorial Africa, 3 Southeast Asia, 4 Canada, 5 Russia, 6 Southwest South America, 7 USA, 8 China, 9 South Asia, 10 South Africa, 11 Northern Africa, 12 Europe, 13 Northern South America, 14 Oceania, 15 Central America, 16 Central Asia, 17 Mideast, 18 Korea and Japan. The data and 18 regions are based on refs [4,57,58].

This study compares the N_2_O emissions and CH_4_ emissions from NTEs of China and other regions/countries, based on the data released by GMB and GNB (Fig. 4). The emissions were calculated using the bottom-up method, as this is the sole method available for obtaining N_2_O emissions from natural soils and CH_4_ emissions from freshwaters [58]. Among the regions, China holds the eighth position, globally, in terms of total GWP arising from N_2_O and CH_4_ from NTEs, which contributes 5.7% of global emissions. Compared with Brazil and Equatorial Africa, which are the top two in terms of total GWP, China's emissions account for approximately half of theirs. Compared with regions in similar latitudinal zones, China's emissions are only lower than those of the USA and higher than those of other regions. Wetland CH_4_ emissions contribute more GWP for China, which is ∼1.5 times that of N_2_O emissions from natural soils. It should be pointed out that the CH_4_ emissions from freshwaters have been taken into account herein, whereas the N_2_O emissions from freshwaters have not been included. CH_4_ uptakes from natural soils only offset ∼10% of the total CH_4_ and N_2_O emissions.

It should be noted that this inventory is designed for a global scale and can be used for comparisons between regions and countries. However, due to methodological and data-related issues—such as the lack of model parameterization and validation specific to China, and overly coarse emission factors—its assessment of China's emissions inevitably contains significant uncertainties.

## LIMITATIONS IN CURRENT INVENTORIES AND COMPREHENSIVE GWP EVALUATION

### Key sources of uncertainty in current inventories

According to our review (Fig. 3), the current Chinese inventories for CH₄ and N₂O emissions from NTEs exhibit significant discrepancies (Fig. 3), due to uncertainties in flux estimates and the extent of NTEs. Among the inventories focusing on China, process-based models generally provide more consistent results than the extrapolation method. However, the deficiencies in current models still introduce errors in flux calculations. Several important processes are underrepresented in current process-based models. For instance, most process-based CH_4_ models (Tables S1 and S2) simulate aerobic CH_4_ oxidation but neglect anaerobic CH_4_ oxidation (AOM), despite evidence of its widespread occurrence [73]. Only the TEM model [47] includes an optional algorithm for AOM, based on redox potential and electron acceptor availability. The Michaelis-Menten equation, commonly used in simulating aerobic CH_4_ oxidation in current models (Table S2), could also be applied to model AOM [74], but it must account for the complexities of AOM, such as the effects of CH_4_ production rate, electron acceptor types and availability, and environmental factors [75]. Another example is the omission of heterotrophic nitrification processes in current N_2_O emission models, despite their unignorable contribution in acid soils [50]. A recently developed model, the IBIS-MicN model [50], has been incorporated into this process, and can serve as a reference for other models.

Moreover, certain environmental factors, such as freeze-thaw cycles and soil salinity, impact vast areas of ecosystems in China but are underrepresented in current CH₄ and N₂O models. This underrepresentation inevitably introduces uncertainties into the flux simulations, particularly in plateau and alpine regions, such as the Qinghai-Tibet Plateau and the Greater and Lesser Khingan Mountains, as well as coastal wetlands. Some models, such as LPJ-wsl [76] and TEM [77] for modeling CH_4_ emissions, as well as TEM [78] and QUINCY [79] for modeling N_2_O emissions, have incorporated the effects of snow and freeze–thaw cycles on soil temperature and moisture, and subsequent GHG emissions. However, complex synergistic effects remain underrepresented in the models. For instance, CH₄ emissions are influenced by the decomposition rates, thawing season and depth of the active layer [80,81], while N₂O emissions during the freeze-thaw period are regulated by factors such as soil moisture, organic matter content, C/N ratio and plant growth [82]. Additionally, newly identified mechanisms, such as higher CH₄ emissions during autumn freeze compared to spring thaw [83] and the role of soil properties in mediating freeze-thaw-related N₂O emissions [84] are also absent in current models. Soil salinity can strongly suppress CH₄ production in coastal wetlands, yet most current CH₄ emission models do not account for this mechanism (Table S1). The CH4MOD_wetland_ model [85] incorporates a linear relationship between salinity and log-transformed CH₄ emissions based on 36 field observations [86]. However, a recent study suggests that multiple factors complicate the generalization of CH₄-salinity relationships [87], emphasizing the need to improve the representation of salinity effects in current models.

In addition to uncertainties in flux simulations, uncertainties in the extent of NTEs also contribute to overall inaccuracies. Firstly, information on the extent of NTEs is often missing in current studies, particularly in inventories of CH₄ uptake and N₂O emissions (Table S5). Although wetland extents have been reported in some studies, significant discrepancies exist due to variations in data sources (Table S5). For example, the reported wetland area ranges from 94 000 to 380 000 km^2^, 90 000 to 305 000 km^2^, and 99 800 to 540 800 km^2^ for the 1990s, 2000s and 2010s, respectively (Table S5). The unclear or inconsistent information of NTEs’ extent has increased the difficulty in evaluating the accuracy of the inventory. Secondly, over the past four decades, the gross forest extent has undergone complex changes, including areas of forest gain, forest loss and a mixture of both [23]. Grassland degradation accounted for 22.7% of China's total grassland area, driven by warm-dry climatic conditions and human activities [24]. Additionally, natural wetlands were significantly converted to croplands before 2000, although some of these converted wetlands have been restored in recent years [25,88]. This land-use change of NTEs has not been fully considered in the national inventories, especially for CH_4_ uptakes (Fig. 3b). Last but not least, current inventories often rely on decadal area data, which inevitably overlook interannual and seasonal variations in GHG emissions. This is particularly true for CH₄ emissions from wetlands, which are heavily influenced by seasonal changes in wetland extent and may introduce significant uncertainties into current inventories [89].

In addition, there are many other areas in current inventories that require improvement. First, few reliable techniques, such as process-based models or ML approaches are available for estimating CH_4_ and N_2_O emissions from freshwaters (Fig. 3d). Second, although the ML methods can disentangle multiple complex interactions between the fluxes and the controlling factors, the demand for massive training data and low interpretability due to the ‘black-box’ use of ML greatly restricts the reality of the upscaled estimations [90]. For example, limited data on CH_4_ uptakes from the forests in southern China constrain national estimates using the ML method [72]. Furthermore, atmospheric variations of CH₄ and N₂O reflect signals from multiple sources and sinks, making flux inversions challenging due to overlapping spatial and temporal patterns. Some inversions rely on prior sectoral contributions within grid cells, while others estimate fluxes from broad categories assuming distinct spatiotemporal distributions, both of which introduce uncertainties. Isotopic signatures in CH₄ and N₂O ratios can help distinguish flux origins [91,92], but their use is limited by sparse observations. With the increasing availability of ground-based and satellite observation data, ML and inversion methods are expected to significantly improve inventory accuracy, though further methodological advancements are still needed.

### Comprehensive evaluation of GWP from CH_4_ and N_2_O in NTEs

Current research on CH₄ and N₂O inventories in Chinese NTEs predominantly relies on a single method, which is significantly behind that of global [6,57] or European budget [93]. Due to the varying strengths and limitations of different methods and models, inventories based on a single approach inherently introduce uncertainties tied to their specific shortcomings. Furthermore, these methods cannot be integrated to comprehensively evaluate the total CH₄ and N₂O emissions and their GWP from NTEs in China.

This study advances the understanding of several scientific uncertainties highlighted above. By integrating multi-model and ML techniques, using consistent input data, and incorporating long-term, high-frequency ecosystem extent data, it provides the latest estimation of CH₄ and N₂O emission inventories from China's natural ecosystems for the period 1980–2020. This inventory offers a more comprehensive perspective on the natural and societal factors influencing CH₄ and N₂O emissions in China's NTEs. Additionally, it facilitates a detailed evaluation of the GWP associated with CH₄ and N₂O emissions from NTEs.

This inventory utilizes three process-based models—CH4MOD_wetland_ [31], TRIPLEXE-GHG [16] and IBIS-CH_4_ [38]—to estimate the CH_4_ emissions from natural wetlands. It also incorporates a regression model [11] and a process-based model (MeMo v1.0) [44] to estimate CH_4_ uptakes from forests, grasslands and shrublands. Additionally, an RF model and two process-based models—TRIPLEXE-GHG [16] and IBIS-MicN [50]—are used to estimate the N_2_O emissions from forests, grasslands and shrublands (Table S5 and Supplementary S1.1). All models have been validated using the site-specific flux observations of CH_4_ emissions from wetlands, CH_4_ uptakes and N_2_O emissions from forests, grasslands and shrublands. The estimations rely on consistent environmental forcing data sets. Annual data sets on the distribution of forests, grasslands and shrublands, along with seasonal wetland distributions, are utilized (Supplementary S3, Table S7). The integrated GWP over a 100-year time horizon (GWP-100) is calculated for CH₄ and N₂O sources and sinks, using their radiative forcing constants relative to CO₂: 27 for CH₄ and 273 for N₂O [2]. A detailed description of the methods and data sets is provided in Supplementary S3.

**Figure 5.**
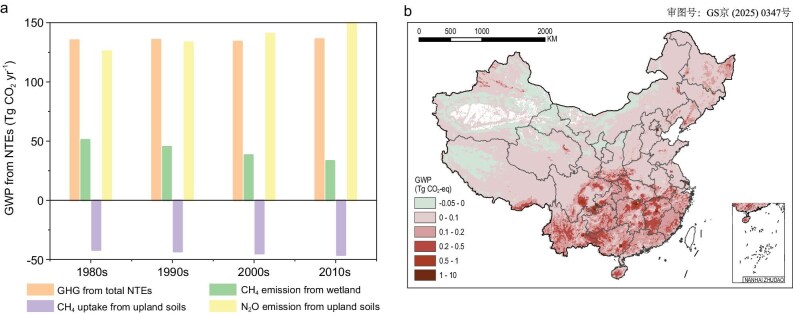
(a) Temporal variations of decadal-mean GWP and (b) spatial variations of cumulative GWP induced by CH_4_ and N_2_O sources and sinks from Chinese NTEs during the 1980s to 2020s.

The new inventory reveals that China's NTEs were significant sources of CH_4_ and N_2_O emissions between 1980 and 2020, with an accumulated GWP of 5.55 Pg CO₂-eq. The comprehensive GWP from CO₂ and N₂O emissions showed no significant temporal trend during this period (Fig. 5a). However, it exhibits a distinct spatial distribution pattern, with weak sources and sinks in North China, Northwest China and the northwestern Qinghai-Tibet Plateau, and significant sources in Northeast and South China (Fig. 5b). The cumulative CH₄ uptake by forests, grasslands and shrublands (−1.81 Pg CO₂-eq) (Fig. S2a) is nearly offset by CH₄ emissions from natural wetlands (1.71 Pg CO₂-eq) (Fig. S2b), rendering the NTEs close to CH₄ neutrality. The net warming effect of NTEs is primarily driven by N₂O emissions, which amount to 5.65 Pg CO_2_-eq (Fig. S2c).

The spatiotemporal variations in the comprehensive GWP from CO₂ and N₂O emissions are influenced by natural factors i.e. climate and land cover distribution, and social factors, including human activities. The spatiotemporal distribution of net GWP reflects the combined effects of CH_4_ sources from wetlands (Fig. S2b), CH_4_ sinks from uplands (Fig. S2a) and N_2_O sources (Fig. S2c). The forests and grasslands in Northwest China exhibit a balance between CH_4_ uptake and N_2_O emissions, resulting in some areas acting as weak sources and others as weak sinks. The CH_4_ emissions from wetlands in Northeast China characterize this region as greenhouse gas sources. The N_2_O emissions from forests in Southeast China outweigh CH_4_ uptake, combined with CH_4_ emissions from wetlands in Hunan, Jiangxi and Sichuan provinces, resulting in Southeast China being a significant greenhouse gas source.

The temporal variation of the net GWP is driven by CH₄ sources and sinks, as well as N₂O sources, influenced by climate change and human activities. The fluxes of wetland CH₄ emissions, CH₄ uptake and N₂O emissions from forests, grasslands and shrublands all exhibit significant increasing trends (Fig. S3a), primarily driven by interactive effects of climatic and environmental factors. For example, rising temperatures (Fig. S3c) enhance methanogenic activity, while increased precipitation (Fig. S3c) expands the flooded areas of seasonal wetlands and raises water levels, contributing to higher CH_4_ emissions [13,94]. Warming and drought enhance CH₄ uptake, but wet conditions weaken this positive effect by altering soil water content [95]. Additionally, atmospheric nitrogen deposition has driven increases in natural soil N₂O emissions [17]. CO₂ fertilization promotes plant growth, supplying more methanogenic substrates and thereby increasing CH₄ emissions [94]. However, it may also reduce CH₄ uptake rates in forest soils [96].

The temporal variations in total national emissions were significantly influenced by human activities, which caused substantial changes in land use (Fig. S3c). Natural wetlands show significant decrease from 1980 to 2020, with a rate of 600 km^2^ yr^−1^ (Fig. S3c). Accordingly, wetland CH_4_ emissions in the 2000s decreased by 0.66 Tg CH_4_ compared to the 1980s (Fig. 5a), at a rate of 0.02 Tg CH_4_ yr^−1^ (Fig. S3a). In contrast, the total area of forests, grasslands and shrublands increased by 12.9 × 10^4^ km^2^, particularly after 2000 (Fig. S3a). As a result, the CH_4_ uptakes and N_2_O emissions respectively increase by 0.16 Tg CH_4_ yr^−1^ and 0.08 Tg N_2_O yr^−1^ (Fig. 5a). The increases and decreases in CH_4_ and N_2_O sources and sinks offset each other, keeping the net GWP of China's ecosystems around 135 Tg CO₂-eq over the past 40 years (Fig. 5a).

This inventory is part of the China GHG Emission Dataset (CNGHG), which includes total anthropogenic and national CO₂, CH₄ and N₂O sources and sinks [97]. However, there are still knowledge gaps in this estimation that need to be addressed and further analyzed in the future.

## IMPLICATIONS FOR RESEARCH AND POLICY-MAKING

This review highlights the knowledge gaps in the current CO₂ and N₂O inventory compilation for China's NTEs. The national inventory compilation significantly lags behind global efforts. Additionally, most inventories are compiled by researchers and are overlooked in the existing NGHGIs. While the Chinese government has released wetland CH₄ emission inventories in its NGHGIs [26,27], substantial discrepancies exist between the third and fourth NGHGIs, likely due to differences in site-specific observations used in the extrapolation methods (Table S5). To date, CH₄ uptake inventories and N₂O emission inventories from NTEs have not been included in the current NGHGIs.

Additionally, given that the non-CO₂ policies in China's NDCs still lack a clear, overarching quantitative target for non-CO₂ mitigation [98], this review offers suggestions for managing natural ecosystems to minimize non-CO₂ emissions. China has been implementing a series of national restoration programs to address the degradation of natural ecosystems, such as afforestation, conversion of cropland to forest, grassland restoration and wetland restoration projects, with plans to continue and expand these efforts in future strategies. Based on the results of this study (Fig. 5b, Fig. S4), these measures, when implemented across different regions and time periods, are likely to result in varying changes in CH₄ and N₂O emissions. Therefore, an integrated planning approach should be adopted, considering not only the carbon peak timeline and restoration costs, but also a comprehensive assessment of GHG emissions and other ecological, social and economic benefits of restoration. On a national scale, net CH_4_ and N_2_O fluxes are lowest in Northwest China, followed by northeast China, while they are highest in South China (Fig. S4). Since grassland restoration has minimal effect on N₂O emissions [99], it is recommended to prioritize grassland restoration in Inner Mongolia, Xinjiang and the Tibetan Plateau, where it can enhance CH_4_ uptake (Fig. S2d) and contribute to biodiversity conservation and sustainable grazing systems. When undertaking wetland restoration, the spatial heterogeneity of CH_4_ fluxes should be considered (Fig. S2e), with priority given to restoring areas with low CH_4_ fluxes, such as the Tibetan Plateau and coastal wetland regions [13]. Restored wetlands can also provide crucial ecosystem services such as water purification, flood control and habitat for wildlife. Ecosystem restoration in southern regions may result in relatively high non-CO₂ emissions (Fig.5b, Fig. S4). However, considering the carbon sequestration capacity of forests and the fact that farmlands generate more N₂O emissions compared to natural ecosystems, priority should be given to converting farmland back to forests along major rivers and in the southwestern mountainous areas. This approach not only mitigates N₂O emissions from agricultural land but also supports long-term environmental resilience and sustainable land-use practices.

## SUMMARY

This review presents the conventional view of major processes, environmental controls of CH_4_ and N_2_O dynamics in NTEs, and reviews the evaluation methodologies of the sources and sinks for both gases. By examining existing studies for Chinese NTEs, this article highlights progress with regard to national CH_4_ and N_2_O inventory complications, and addresses major uncertainties in current inventories. Furthermore, this study addresses several issues in current estimations by using a multi-model approach to comprehensively evaluate CH₄ and N₂O emissions from China's NTEs over the past 40 years. It provides a clearer understanding of the spatiotemporal patterns of the integrated GWP of CH₄ and N₂O in China's NTEs and reveals the impacts of climate change and land-use changes on these emissions. This review highlights the oversight of CH₄ and N₂O emissions from NTEs in NGHGIs and offers recommendations for enhancing sinks and reducing emissions of CH₄ and N₂O from NTEs.

## Supplementary Material

nwaf094_Supplemental_File

## Data Availability

The new non-CO_2_ GHG budget generated in this study using NTEs is available at http://carbon.pku.edu.cn.
