# Supplementary material for: Methane and nitrous oxide budget for Chinese natural terrestrial ecosystems
Source: Natl Sci Rev. 2025 Mar 11;12(4):nwaf094. doi: 10.1093/nsr/nwaf094 (PMC11974395; doi:10.1093/nsr/nwaf094)
Supplement: nwaf094_Supplemental_File [file nwaf094_supplemental_file.docx]

**Supplementary Information for Methane and nitrous oxide budget from Chinese natural terrestrial ecosystems**

Tingting Li^1, 2 *^, Xinyi Liu^1,3^, Jiahui Tian^1,3^, Wenping Yuan^2, 4^, Xuhui Wang^4^, Xiu-qun Yang^5^, Songbai Hong^6^, Yilong Wang^7^, Qiuan Zhu^8^, Lijun Yu^9^, Jiangzhou Xia^10^, Han Xiao^11^, Minqi Liang^11^, Shihua Li^11^, Zimeng Li^4^, Yingxuan Wang^4^, Kerou Zhang^12^, Min Xu^8^, Zhangcai Qin^2,11^

1 Key Laboratory of Atmospheric Environment and Extreme Meteorology, Institute of Atmospheric Physics, Chinese Academy of Sciences, Beijing, China

2 International Research Center of Big Data for Sustainable Development Goals

3 College of Earth and Planetary Sciences, University of Chinese Academy of Sciences, Beijing, China

4 Institute of Carbon Neutrality, Sino-French Institute for Earth System Science, College of Urban and Environmental Sciences, Peking University, Beijing, China

5 School of Atmospheric Sciences, Nanjing University, Nanjing, China

6 School of Urban Planning and Design, Shenzhen Graduate School, Peking University, Shenzhen, China

7 State Key Laboratory of Tibetan Plateau Earth System, Resources and Environment

(TPESRE), Institute of Tibetan Plateau Research, Chinese Academy of Sciences,

Beijing, China

8 College of Geography and Remote Sensing, Hohai University, Nanjing, China

9 Aerospace Information Research Institute, Henan Academy of Sciences, Zhengzhou,

China

10 Tianjin Key Laboratory of Water Resources and Environment, Tianjin Normal

University, Tianjin, China

11 School of Atmospheric Sciences, Guangdong Province Data Center of Terrestrial

and Marine Ecosystems Carbon Cycle, Sun Yat-sen University, Zhuhai, Guangdong,

China

12 Wetland Research Center, Institute of Ecological Conservation and Restoration,

Chinese Academy of Forestry, Beijing, China

* Correspondence should be addressed to: Tingting Li ([litingting@mail.iap.ac.cn](mailto:litingting@mail.iap.ac.cn))

**S1. Major processes and controlling factors**

**S1.1 Pathways of microbiological methanogenesis**

The microbiological methanogenesis appears through three pathways: acetoclastic, hydrogenotrophic and methylotrophic methanogenesis. In acetoclastic methanogenesis, acetate is dismutated by acetotrophic methanogens to CO_2_ and CH_4_. In hydrogenotrophic methanogenesis, hydrogenotrophic methanogens utilize H_2_ as an electron donor for the reduction of CO_2_ to produce CH_4_. Previous studies have suggested that the acetoclastic methanogenesis and hydrogenotrophic methanogenesis are primary pathways, which account for 50–90% and 10–43% of global annual CH_4_ produced, respectively [1]. Recently, methylotrophic methanogenesis, in which the methylated compounds are reduced to CH_4_, has been considered to be widespread in anaerobic environment than previously thought [2].

The aerobic and anaerobic oxidations are two important pathways of microbial CH_4_ oxidation [3]. Under the aerobic conditions, the methanotrophs oxidize CH_4_ sequentially to methanol, formaldehyde, formate and finally CO_2_ [4]. The enzyme methane monooxygenase (MMO) catalyzes this reaction with oxygen as the terminal electron acceptor. The ‘high affinity oxidation’ and ‘low affinity oxidation’ are two forms of aerobic CH_4_ oxidation, which occur at CH_4_ concentration <12 ppm and >40 ppm, respectively [5]. There are two forms of MMO. A particulate methane monooxygenase (pMMO) occurs in almost all methanotrophs, while a soluble form (sMMO) has a more restricted distribution [4]. CH_4_ oxidation also appears in the anaerobic conditions. The anaerobic methanotrophic archaea mediates this process through the coupling of CH_4_ oxidation to different electron acceptors, or in concert with a syntrophic bacterial partner [6]. According to the used electron acceptor, the anaerobic CH_4_ oxidation can be divided into nitrogen-, sulfate- and metal- dependent oxidations [7].

**S1.2 Controlling factors of CH_4_ cycling**

The soil temperature and pH could affect microbial activities of methanogens and methanotrophs [4]. For upland soils, the soil moisture shows a complex relationship with CH_4_ production and consumption [8]. Extremely high soil moisture may reduce soil porosity and O_2_, inhibiting the diffusion of CH_4_ from the atmosphere to the soil layer, while extremely low soil moisture can limit biological activity of methanotrophs [9]. For wetlands, the water table depth is an important controlling factor, since it determines the aerobic and anaerobic zones for CH_4_ production and oxidation, respectively [10]. The climatic factors such as temperature and precipitation have indirect influences on CH_4_ fluxes through affecting the soil temperature, moisture and water table depth [11]. In addition, plants play vital roles in CH_4_ fluxes, since they not only provide methanogenic substrates via root substrates and litterfall, but also act as a pathway for CH_4_ transport [12][37].

**S1.3 Pathways of N_2_O production**

In the denitrification process, N_2_O can be produced from the stepwise reduction of nitrate (NO_3_^−^) to nitrite (NO_2_^−^), nitric oxide (NO) and N_2_O under anaerobic conditions [13]. The nitrification process could provide NO_3_^−^ as the substrate for denitrification through reducing ammonium (NH_4_^+^), ammonia (NH_3_) or organic N to hydroxylamine (NH_2_OH), NO_2_^−^ and NO_3_^−^ [13]. The intermediate NH_2_OH can also be directly converted to N_2_O or combined with NO thus obtaining N_2_O [13]. The nitrifier denitrification describes the oxidation of ammonia to nitrite and its subsequent reduction to form N_2_O through the same autotrophic ammonia oxidizing organism under the conditions of high N availability but low organic carbon (C) and oxygen availability [14]. The DNRA driven by some specific microorganisms is the dissimilatory reduction of NO_3_^−^ to NO_2_^−^ and NH_4_^+^, with N_2_O formation at the NO_2_^−^ reduction stage [15]. There is also evidence that the N_2_O production is correlated with plant leaf nitrate assimilation activity, which is still an unrecognized process [16]. Besides, the chemodenitrification is an abiotic pathway to form N_2_O, which differs from the above biotic pathways. The chemodenitrification typically involves the reduction of NO_2_^−^ via Fe (II), resulting in the production of N_2_O, which is generally stimulated in the environments where oxygen level decreases and the concentrations of Fe (II) and organic matter increase [17].

**S1.4 Non-biological controlling factors of N_2_O production and consumption**

The soil moisture controls the denitrification by regulating anaerobic environment [18]. The temperature and soil moisture could also affect the microbial reaction activities of the biological processes [18]. The soil temperature lower than −7°C may significantly reduce the soil microbial activities and N_2_O emission [19]. The soil pH can regulate N_2_O emission by influencing biotic and abiotic processes. Specifically, the soil pH is positively related to N_2_O production through nitrifier denitrification, and the acidic soil stimulates the chemodenitrification process [18]. The elevated nitrogen deposition could provide more substrates and thus increase N_2_O emission [20]. Besides, the carbon availability and soil type could also directly or indirectly influence N_2_O emission [21].

**S2. Methodologies for CH_4_ and N_2_O budget estimation**

**S2.1** **Data-driven method**

The empirical extrapolation method extrapolates the field flux measurements, i.e., flux observations by chamber or eddy covariance technique, into a given area to get the regional GHG emissions. It follows the Tier 1 or 2 method recommended by the Intergovernmental Panel on Climate Change (IPCC) for the National Inventory, and is popular in early stage with rare observations. This approach usually induces great uncertainties due to the spatial heterogeneity of GHG fluxes, and therefore is not recommended.

The statistical models establish the empirical equations between the fluxes and multiple environmental factors based on abundant field observations. For example, Yu et al. [8] developed empirical equations between CH_4_ uptake flux and climatic soil controls to estimate the CH_4_ uptake from global forests and grassland soils. The gray-box model is a typical statistical model, which deduces a linear relationship between soil N_2_O emission and N deposition [20] . The statistical model may be appropriate for the estimations at a large spatial scale and/or a long temporal scale, where/when the N or C cycling process related parameters tend to stabilize and are mainly determined by ecosystem state factors. However, they still may result in some uncertainties, as the mechanistic processes such as GHG production, consumption and transport are ignored.

The machine learning (ML) has been increasingly applied to estimate regional CH₄ and N₂O budgets. For example, Peltola et al. [22] used RF to upscale eddy covariance fluxes for CH₄ emissions from northern wetlands, while McNicol et al. [23] developed a six-predictor RF model for a global CH₄ inventory based on FLUXNET-CH4 data. Similarly, Liao et al. [24] produced a global map of terrestrial soil N₂O emissions using RF.

**S2.2 Development of the atmospheric inversion method**

Secular observations of GHG mole fractions in the atmosphere began in the 1950s. The program of National Oceanic and Atmospheric Administration to measure atmospheric CH_4_ through its global cooperative flask sampling network started in 1983 [25] and now includes more than 150 sites (https://gml.noaa.gov/dv/site/index.php?program=ccgg). Observations of CH_4_ from space began with the SCIAMACHY instrument since 2003 and were continued with the TANSO-FTS instrument aboard the GOSAT, the TROPOMI instrument aboard Sentinel-5 Precursor, MethaneSat and many other missions [26]. During the last decade, satellite observations have provided a new data stream for atmospheric inversions and new insights into the distribution and magnitude of CH_4_ fluxes from facility to global scales. However, current N_2_O space-borne observations are mainly performed in the thermal infrared spectral region which is sensitive to N_2_O in the middle-upper troposphere (around 300 hPa) rather than the lower troposphere where the mixing ratios are mostly affected by land-atmosphere fluxes.

**S3. Description of methods and data for the new inventory**

This new CH_4_ and N_2_O inventory from natural terrestrial ecosystems (NTEs) is included in the China GHG emission dataset (CNGHG) inventory [27]. It used 3 process-based models (CH4MOD_wetland_ [28, 29], TRIPLEXE-GHG [30] and IBIS-CH_4_ [31]) to estimate CH_4_ emissions from natural wetlands, a regression model [8] and a process-based model (MeMo v1.0 [32]) to estimate CH_4_ uptakes from forests, grasslands and shrublands, as well as a random forest (RF) model [33] and two process-based model (TRIPLEXE-GHG [30] and IBIS-MicN [34, 35]) to estimate N_2_O emissions from forests, grasslands and shrublands, respectively.

For the estimation of CH_4_ emissions from natural wetlands, three models were firstly used to simulate the annual CH_4_ fluxes in each grid based on the unified environmental drivers (Table S7). CH4MOD_wetland_ is a biogeophysical model to simulate CH_4_ production, oxidation, and emission processes from natural wetlands [28]. The model inputs include soil temperature, annual net primary productivity (NPP), water table depth, soil sand fraction, bulk density and soil organic carbon content (SOC). The model separates vegetation type as vascular vegetation, shrub and forest. It has been completely calibrated and validated across Chinese natural wetlands, including 2 sites in Northeast China, four sites on the Qinghai Tibet Plateau, one site in Inner Mongolia and two sites in the Eastern China [11, 36]. IBIS-CH_4_ is a fully coupled process-based model including three major processes of production, oxidation, and transport, and especially simulates microbial dynamics [31]. Inputs of the model include multi-layer soil temperature, leaf area index (LAI), soil PH, SOC, bulk density and water table depth. It has been validated against two sites in Northeast China and two sites on the Qinghai Tibet Plateau [31]. TRIPLEX-GHG is a dynamic global vegetation model and a process-based wetland CH_4_ emission module was integrated with considering the processes of production, consumption, three ways of transport for wetland CH_4_, as well as the process of dynamic water table and a specific plant function type for wetland [30]. The inputs TRIPLEX-GHG include air temperature, precipitation, radiation, relative humidity and water table depth. It has been validated against one site in Northeast China and six sites on the Qinghai Tibet Plateau. All of three models used the water table depth as a driving forcing, which was simulated by TOPMODEL [37].

The estimation of CH_4_ uptakes from natural terrestrial ecosystems (NTE) were based on Yu’s regression model [8] and MeMo v1.0 [32]. Yu’s model is an empirical relationship between the CH_4_ uptake fluxes and meteorological and soil factors based on an analysis of observed data from globally distributed sites. The model inputs include annual mean air temperature, annual precipitation, soil sand, silt and clay fraction, SOM and soil PH. The model runs on an annual step, and is suitable for forests and grasslands on a global scale [8]. The MeMo model (v1.0) is a process-based model that simulates atmospheric CH_4_ uptake by soils, parameterized using globally observed data. Inputs include soil temperature, soil moisture, atmospheric CH_4_ concentration, nitrogen deposition, nitrogen fertilizer input, paddy field distribution, land use, soil bulk density and clay content, all interpolated to a 0.1° spatial and monthly temporal resolution. The model has been validated against both global estimates and observational data, demonstrating high accuracy in predicting soil CH_4_ uptake [32].

Two process-based models, TRIPLEXE-GHG [30] and IBIS-MicN [34], as well as one random forest model were used to estimate N_2_O emissions from natural terrestrial ecosystems. The TRIPLEX-GHG model incorporates a submodule specifically designed to simulate N_2_O fluxes by integrating the processes of nitrification, denitrification, and diffusion [38]. The nitrification rate is modeled using Monod kinetics, while a double substrate-based (DOC and NOx) Michaelis–Menten equation is employed to simulate the relative growth rate of denitrifiers at each step. The Pirt equation is utilized to calculate the consumption rates of NO_3_^-^, NO_2_^-^, NO, and N_2_O during denitrification. The model-driven data was the same as applied in wetland CH_4_ simulation. The model has been calibrated and validated on a global scale. The IBIS-MicN model simulates N_2_O emissions from four N_2_O-producing processes, i.e., autotrophic nitrification, heterotrophic nitrification, nitrifier denitrification, and denitrifier denitrification [35]. Meanwhile, the dynamic activities of nitrifiers and denitrifiers, as well as the ammonium and nitrate contents in the soil, were simulated in the IBIS-MicN model. The model inputs include meteorological (air temperature, precipitation, cloud fraction, wind speed, air pressure, and relative humidity) dataset, N deposition, and soil pH. The IBIS-MicN model has been validated by observation data from 23 forest and grassland validation sites in China with R^2^ being 0.85 [34]. N_2_O fluxes from NTE were also estimated utilizing RF models based on a dataset of N_2_O emission flux records [33]. This dataset comprised 184 measurements from forests and 135 from grasslands and shrublands, sourced from 212 papers. The RF models incorporated input variables such as mean annual temperature, mean annual precipitation, soil pH, bulk density, soil total nitrogen, C: N, leaf area index, and nitrogen deposition. To prevent overfitting, the measurement records were randomly split into two sets, with 80% allocated for training the models and the remainder reserved for testing purposes.

All the models were listed in Table S6. Most of them followed the parameterization of previous studies, since they have upscaled to the national scale [8, 11, 34, 39]. More details about the model description, calibration and validation were described in previous studies [11, 31, 40].

To drive ecosystem models and data-driven models, we need a long-term series of meteorological, soil, vegetation, hydrological dataset. Table S7 listed the description, sources and application of the datasets. Upscaling was completed by rasterizing the inputs with 0.1°× 0.1°. All the models were driven by the 0.1°× 0.1° datasets to compute the CH_4_ and N_2_O uptake and emission fluxes. Then, we separately calculated the mean fluxes of multiple models in each grid for wetland CH_4_ emission, CH_4_ uptake and N_2_O emission from NTE, respectively. Next, we combined the CH_4_ and N_2_O fluxes with areas of NTE to evaluate the emissions. Finally, we calculated an integrated global warming potential at a 100-year time zone (GWP-100) from CH_4_ and N_2_O sources and sinks based on their radiative forcing constants of CH_4_ (27) and N_2_O (273) relative to CO_2_ [41].

**Table S1** Description of wetland CH_4_ emission models involved in Global Methane Budget

| Model | Description | Reference |
| --- | --- | --- |
| CH4MOD_wetland_ | An independent biogeophysical process-based model with methanogenic substrates from root exudates and the decomposition of litter and soil organic matter (SOM). Involve plant-mediated transportation, ebullition and diffusion. | [28, 29] |
| CLASS-CTEM | The model includes representations of dynamic natural wetlands and their CH_4_ emissions, CH_4_ emissions from fires, and uptake of CH_4_ by soils. The heterotrophic respiration (Rh) is scaled to account for CH_4_ vs. CO_2_ emitted and differences in upland vs. lowland Rh. No specific transport pathways. | [42] |
| DLEM | A highly integrated process-based ecosystem model that aims at simulating the fluxes and storages of carbon, water and nitrogen among/within terrestrial ecosystem components. The methanogenic substrates are derived from the dissolved organic carbon. Involve plant-mediated transportation, ebullition and diffusion. | [43] |
| ELM-ECA | A biogeochemistry model integrated in the Community Climate System Model (CCSM4) and the Community Earth System Model (CESM1). The CH_4_ production in the anaerobic portion of the soil column is related to the Rh. Involve plant-mediated transportation, ebullition and diffusion. | [44] |
| ISAM | The model contains a biogeophysical component, a dynamic vegetation component and a soil biogeochemistry component. The CH_4_ production is linked to Rh. Involve plant-mediated transportation, ebullition and diffusion. | [45] |
| JSBACH | The CH_4_ production depends on anoxic respiration produced by YASSO soil carbon model modified to account for anoxic conditions and coupled to JSBACH. Involve plant-mediated transportation, ebullition and diffusion. | [46] |
| JULES | The joint UK land environment simulator to model energy, water and carbon fluxes. The CH_4_ production is linked to the net primary productivity (NPP) or soil carbon. No specific transport pathways。 | [47] |
| LPJ-GUESS | A model that simulates plant physiology, carbon allocation, decomposition, hydrological fluxes and wetland CH_4_ fluxes. The methanogenesis is linked to the decomposition of litter and soil organic carbon. Involve plant-mediated transportation, ebullition and diffusion for the regions with latitude higher than 40°N. | [48] |
| LPJ-MPI | A coupled model of CLIMBER2 and DGVM LPJ, with modules to simulate wetland extent, peat accumulation and CH_4_ fluxes; The CH_4_ production is linked to Rh. Involve plant-mediated transportation, ebullition and diffusion. | [49] |
| LPJ-WSL | A process-based dynamic global vegetation model based on development of the LPJ-DGVM. The methanogenesis is linked to NPP and the decomposition of SOM. No specific transport pathways. | [50] |
| LPX-Bern | A DGVM model coupled water, nitrogen and carbon cycles. The methanogenesis is linked to NPP and the decomposition of SOM. Involve plant-mediated transportation, ebullition and diffusion. | [51, 52] |
| ORCHIDEE | ORCHIDEE simulates the land energy, hydrology and the carbon cycle, with a module to simulate wetland CH_4_ fluxes. CH4 substrates are derived from NPP and the decomposition of SOM. Involve plant-mediated transportation, ebullition and diffusion. | [53] |
| SDGVM | A generalized, global-scale model that predicts vegetation structure and dynamics. The methanogenic substrates are from decomposition of SOM. No specific transport pathways | [54, 55] |
| TEM-MDM | A methane module coupled to a process-based biogeochemistry model (TEM); CH_4_ production is modeled as an anaerobic process that occurs in the saturated zone of the soil profile, controlled by methanogenic substrate availability, soil temperatures, PH, and redox potential. Five primary types of wetlands are considered. Involve plant-mediated transportation, ebullition and diffusion. | [56, 57] |
| VISIT | A process-based biogeochemical model with methanogenic substrates related with NPP and the decomposition SOM. Involve plant-mediated transportation, ebullition and diffusion. | [58] |
| TRIPLEX-GHG | A CH_4_ emission model integrated into a dynamic global vegetation model (DGVM) of the Integrated Biosphere Simulator (IBIS). Carbon substrate supply from decomposition of litter and SOM; Involve plant-mediated transportation, ebullition and diffusion. | [30, 40] |

**Table S2** Description of CH_4_ uptake models

| Model | Description | Reference |
| --- | --- | --- |
| Potter’s model | A reaction-diffusion model with a one-dimensional diffusion-reaction equation based on Fick’s first law. | [59] |
| Ridgwell’s model | Develop of Potter’s model by incorporating the activity of methanotrophs. | [60] |
| Curry’s model | Based on reaction-diffusion function and futhur enabled the complete consumption of CH_4_ within the soil profile and used a homogenous Neumann condition at the lower model boundary. | [61] |
| Memov1.0 | Provide a general analytical solution to the diffusion-reaction equation by setting up a threshold for the soil CH_4_ concentration to determine the upper/lower boundary of microbial CH_4_ oxidation, and introduced scalar modifiers to refine the modeled soil moisture, temperature, and N deposition effects on methanotrophy. | [32] |
| LPJ-WHyMe | Adopt the scheme of the Curry’s model, with environmental constraint factors. | [52] |
| VISIT | Integrates the Curry’s model to determine CH_4_ fluxes from different upland soils. | [62] |
| DLEM | Consider three pathways include atmospheric CH_4_ oxidation, CH_4_ oxidation during plant-mediated transport and Soil pore water CH_4_ oxidation. | [43] |
| TRIPLEX-GHG | Adopt the Michaelis–Menten equation and constrained by soil temperature and soil redox potential. | [30] |
| TEM | Employed the Michaelis–Menten equation and only considers the CH_4_ concentration when estimating the CH_4_ oxidation rate. | [63] |
| CLM4Me | Uses double Michaelis-Menten kinetics to describe heterotrophic methanotrophy and depends on both the CH_4_ and O_2_ concentrations. | [44] |

**Table S3** Description of N_2_O emission models

| Model | Description | Reference |
| --- | --- | --- |
| Gray-box model | A conceptual model (a process-augmented data-driven approach);  Deduce a linear relationship between N_2_O emissions and N deposition (low-level N input) in forest soils on large scales;  Assume stable parameters of N cycling process. | [64] |
| DAISY | A simplistic dynamic model for agro-ecosystems;  Simulate nitrification and denitrification rates restricted by environmental variables;  Lack the integration of major N_2_O emission processes. | [65] |
| CRISP-nit | A simplistic model for the nitrogen cycle in a crayfish-rice integrated aquacultural farming system;  High N fertilizer inputs in the rice-fields;  Lack the integration of major N_2_O emission processes. | [66] |
| CASA | A process model coupled ecosystem production and soil carbon-nitrogen on a 1° global grid;  Simulate monthly N_2_O production based on the predicted gross N mineralization and soil moisture. | [67] |
| DAYCENT | Daily time-step version of the CENTURY model;  Simulate N fluxes among atmosphere, plant and soil;  Involve the sub-models of N gas emissions from nitrification and denitrification. | [68] |
| DNDC | A detailed biogeochemical process-based model applicable to agricultural systems, wetlands and forests;  Improve the nitrification and denitrification simulation scheme using a kinetic scheme named ‘anaerobic balloon’, driven by soil redox potential and oxygen availability. | [69, 70] |
| DLEM | A highly integrated process-based model;  Simulate the N fluxes and storage with the consideration of land use, management and disturbances, e.g. fire;  Include several important processes responsible for N_2_O emission, e.g. nitrification, denitrification, but not heterotrophic nitrification and nitrifier denitrification. | [43] |
| IBIS-MicN | A comprehensive process-based N_2_O emission model;  Involve microbial dynamics and intermediate products;  Consider four major N_2_O production pathways, i.e. autotrophic nitrification, heterotrophic nitrification, nitrifier denitrification and denitrifier denitrification | [35] |

**Table S4 Description of Atmospheric CH_4_ inversion models**

| Model | Description | Reference |
| --- | --- | --- |
| CarbonTracker Europe CH_4_ | It uses the TM5 to simulate the atmospheric transport and chemical reactions;  The optimization is made in 34 regions using Ensemble Kalman Filter;  Biogenic and anthropogenic emissions are optimized separately. | [71] |
| GELCA | It uses NIES-TM coupled with FLEXPART to simulate the atmospheric transport and chemical reactions;  The optimization is made in 42 land regions and 1 ocean region using Kalman Smoother. | [72] |
| LMDz-PYVAR | It uses LMDz to simulate the atmospheric transport and chemical reactions;  The optimization is made on each grid cell at a spatial resolution of 3.75° longitude × 1.9° latitude;  It is a variational inversion system that uses the M1QN3 algorithm to minimize the cost function;  It optimizes total CH_4_ fluxes. | [73] |
| MIROC4-ACTM | It uses MIROC4-ACTM to simulate the atmospheric transport and chemical reactions;  The optimization is made on in 54 land regions and 30 ocean regions using the analytical inversion method;  It optimizes the total CH_4_ fluxes. | [74] |
| NICAM-TM | It uses NICAM-TM to simulate the atmospheric transport and chemical reactions;  The optimization is made on each grid cell at a spatial resolution of about 240 km;  It is a 4D-Var inversion system and uses the POpULar scheme to minimize the cost function. | [75, 76] |
| NIES-TM-FLEXPART | It uses NIES-TM coupled with FLEXPART to simulate the atmospheric transport and chemical reactions;  The optimization is made on each grid cell at a spatial resolution of 0.1°;  It is a variational inversion system that uses the M1QN3 algorithm to minimize the cost function. | [77] |
| TM5-CAMS | It uses TM5 to simulate the atmospheric transport and chemical reactions;  The optimization is made on each grid cell at a spatial resolution of 3° longitude × 2° latitude;  It is a variational inversion system that uses the M1QN3 algorithm to minimize the cost function;  Four categories of emissions, namely wetlands, rice, biomass burning, and others, are optimized separately. | [78] |
| TOMCAT | It uses TOMCAT to simulate the atmospheric transport and chemical reactions;  The optimization is made in 5 regions using the analytical inversion method;  Seven categories of emissions, namely agriculture (excluding rice), biomass burning, energy, rice, waste, wetlands, and others, are optimized separately. | [79] |
| GONGGA-CH4 | It uses GEOS-Chem to simulate the atmospheric transport and chemical reactions;  The optimization is made on each grid cell at a spatial resolution of 2.5° longitude × 2° latitude;  It is a variational inversion system that uses the NLS-4DVar algorithm to minimize the cost function;  Five categories of emissions, namely fossil fuel, wetlands, agriculture and waste, biomass burning, and others, are optimized separately. | [80] |

**Table S5**. CH_4_ and N_2_O inventories from natural terrestrial ecosystems in China

| Period | GHG (Tg yr^-1^) | Area (M ha) | References |
| --- | --- | --- | --- |
| CH_4_ emissions from natural wetlands | | | |
| 1988 | 1.70 | NAN | [81] |
| 1980 | 2.30 | 13.00 | [82] |
| 1980 | 3.50 | 17.40 | [83] |
| 1980 | 2.04 | 19.96 | [39] |
| 1980s | 4.56 | NAN | [84] |
| 1988-2000 | 2.20 | NAN | [85] |
| 1996 | 2.00 | 18.70 | [86] |
| 1990s | 10.5 | 38.00 | [87] |
| 1990 | 6.65 | 35.50 | [88] |
| 1990 | 2.00 | 11.00 | [82] |
| 1990 | 3.03 | 13.54 | [83] |
| 1990 | 2.46 | 18.91 | [39] |
| 1990s | 3.99 | NAN | [84] |
| 1995-2004 | 1.76 | 9.40 | [89] |
| 2000 | 1.90 | 9.40 | [82] |
| 2000 | 5.71 | 30.50 | [88] |
| 2000 | 3.15 | NAN | [90] |
| 2000 | 2.17 | 10.62 | [83] |
| 2000 | 1.59 | 13.70 | [39] |
| 2000s | 3.86 | NAN | [84] |
| 2000s | 6.67±0.6 | NAN | [91] |
| 2000s | 3.06±0.63 | NAN | [91] |
| 2003-2009 | 4.76 | 9.00 | [92] |
| 2008 | 1.91 | 14.44 | [39] |
| 2010 | 2.15 | 9.98 | [83] |
| 2010 | 1.74 | 40.00 | [93] |
| 2017 | 3.98 | 54.08 | [94] |
| 2005-2018 | 25.25 | 53.42 | [95] |
| 2010s | 7.24±4.28 | NAN | [91] |
| 2010s | 3.20±0.71 | NAN | [91] |
| CH_4_ uptakes from NTEs excluded freshwaters | | | |
| 1961-2005 | -1.07 | NAN | [96] |
| 2000s | -2.33 | NAN | [88] |
| 2000s | -1.32 | NAN | [97] ^*^ |
| 2000s | -1.87 | NAN | [84] |
| 2000s | -2.05±0.17 | NAN | [91] |
| 2000s | -1.83±0.19 | NAN | [91] |
| 2010s | -2.08±0.17 | NAN | [91] |
| 2010s | -1.84±0.23 | NAN | [91] |
| N_2_O emissions from NTEs excluded freshwaters | | | |
| 1961-2005 | 0.70 | NAN | [96] |
| 1980s | 0.94±0.63 | NAN | [98] |
| 1980s | 0.34 | NAN | [34] ^*^ |
| 1980s | 0.25 | 487.00 | [99] ^*^ |
| 1990s | 1.26±0.63 | NAN | [98] |
| 1990s | 0.37 | NAN | [34] ^*^ |
| 1990s | 0.27 | 487.00 | [99] ^*^ |
| 1994-1995 | 0.65 | 181.10 | [100] ^*^ |
| 2000s | 1.32 | NAN | [88] |
| 2000s | 1.04 | 722.30 | [101] |
| 2000s | 0.30 | 487.00 | [99] ^*^ |
| 2000s | 1.57±1.26 | NAN | [76] |
| 2000s | 0.37 | NAN | [34] ^*^ |
| 2007-2016 | 1.57±1.57 | NAN | [76] |
| 2010s | 0.30 | 487.00 | [99] ^*^ |
| 2010s | 0.37 | NAN | [34] ^*^ |
| CH_4_ emissions from freshwaters | | | |
| 2000s | 0.46±0.13 | NAN | [90] |
| 2000s | 5.23 | NAN | [91] |
| 2010s | 2.08±0.59 | 10.91 | [102] ^^^ |
| 2010s | 5.23 | NAN | [91] |
| N_2_O emissions from freshwaters | | | |
| 2010s | 0.13 | 10.91 | [102] ^^^ |

^*^ The budget is for forests and grasslands

^^^ Include lakes and ponds

NAN means no information reported in the publications

**Table S6**. Description of the models used in this study

| Name | Type | Output | Step | Reference |
| --- | --- | --- | --- | --- |
| CH4MOD_wetland_ | Process-based model | CH_4_ emission | daily | [28] |
| IBIS-CH_4_ | Process-based model | CH_4_ emission | daily | [31] |
| TRIPLEXE-GHG | Process-based model | CH_4_ and N_2_O emission | daily | [30] |
| IBIS- MicN | Process-based model | N_2_O emission | daily | [35] |
| RF model | Data driven | N_2_O emission | annual | [33] |
| Memo V1.0 | Process-based model | CH_4_ uptake | monthly | [32] |
| Yu’s model | Data driven | CH_4_ uptake | annual | [8] |

**Table S7**. Description of the driving datasets

| Datasets | Description | Source |
| --- | --- | --- |
| Meteorological data | Daily meteorological data from 1980 to 2020, 0.1º×0.1º (Temperature, Precipitation, longwave radiation, shortwave radiation, Pressure, Specific Humidity, Maximum temperature, Minimum temperature, Dewpoint temperature, Wind speed) | [103] |
| Soil temperature | Daily soil temperature at four layers from 1980 to 2020, 0.1º×0.1º. | [103] |
| Soil moisture | Daily soil moisture at four layers from 1980 to 2020, 0.1º×0.1º. | [103] |
| Soil organic carbon content | Constant value at 6 soil layers, 0.1º×0.1º | [104] |
| Soil PH | Constant value at 6 soil layers, 0.1º×0.1º | [104] |
| Bulk density | Constant value at 6 soil layers, 0.1º×0.1º | [104] |
| Soil texture | Constant value at 6 soil layers, 0.1º×0.1º | [104] |
| Water table | Dail water table depth from 1980 to 2020, 0.1º×0.1º. Simulated by TOPMODEL, driving by soil moisture and CTI. | [105] |
| NPP | 8-day GLASS NPP, 1980 to 2020, 500-m×500-m. | [82] |
| LAI | 8-day GLASS LAI data, from 1980 to 2020, 0.1º×0.1º (V6) | http://www.glass.umd.edu/LAI/MODIS/0.1D/ |
| Wetland distribution | Reconstructed national monthly wetland dynamics based on WAD2M from 2000 to 2020, through random forest model driving by meteorological data on a grid scale. Superimposed the wetland conversion by adding the RF-based wetland seasonalities and the recent wetland loss data based on Fluet-Chouinard to obtain the monthly wetland dynamics from 1980 to 2020. Constrained the provincial area by the third national soil census data. | [103, 106, 107] |
| Forest, grassland and shrubland distribution | The annual LUCC dataset from 1980 to 2020 was produced through fusion of the Chinese Forest Cover Dataset (CFCD) and China Land Cover Data (CLCD). | [108] |


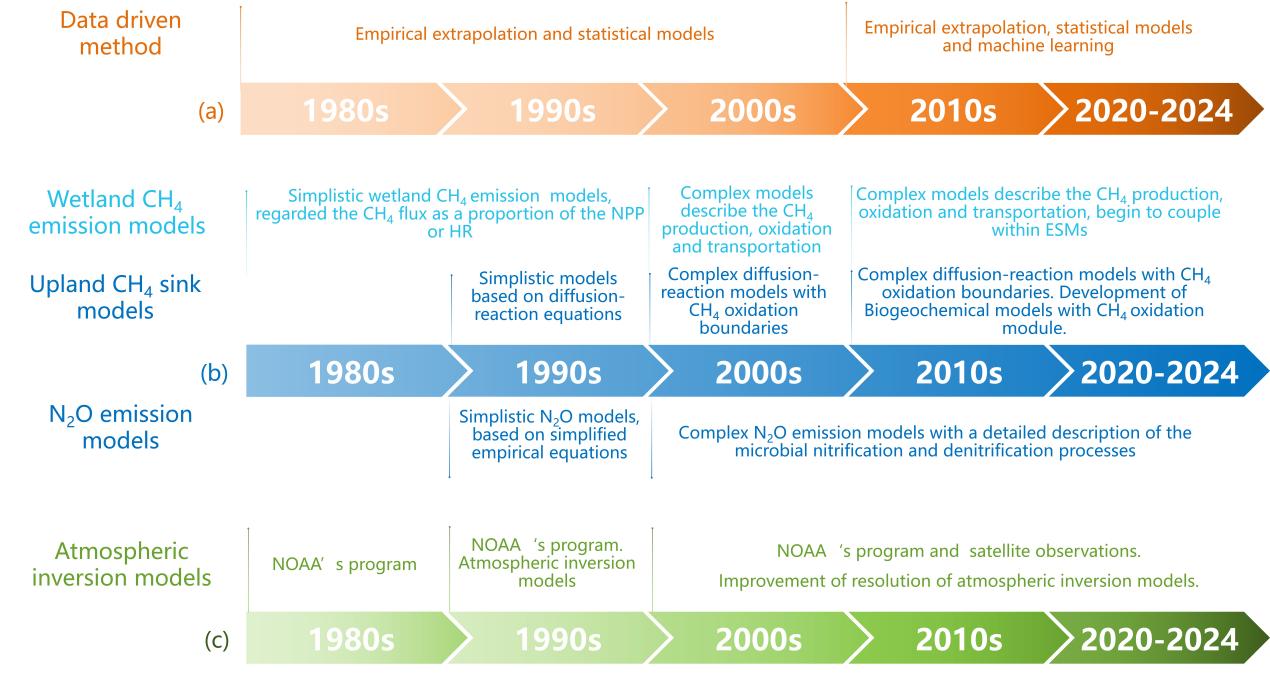


Fig. S1 Evolutions of (a) data-driven method, (b) process-based models and (c) atmospheric inversion models.

**
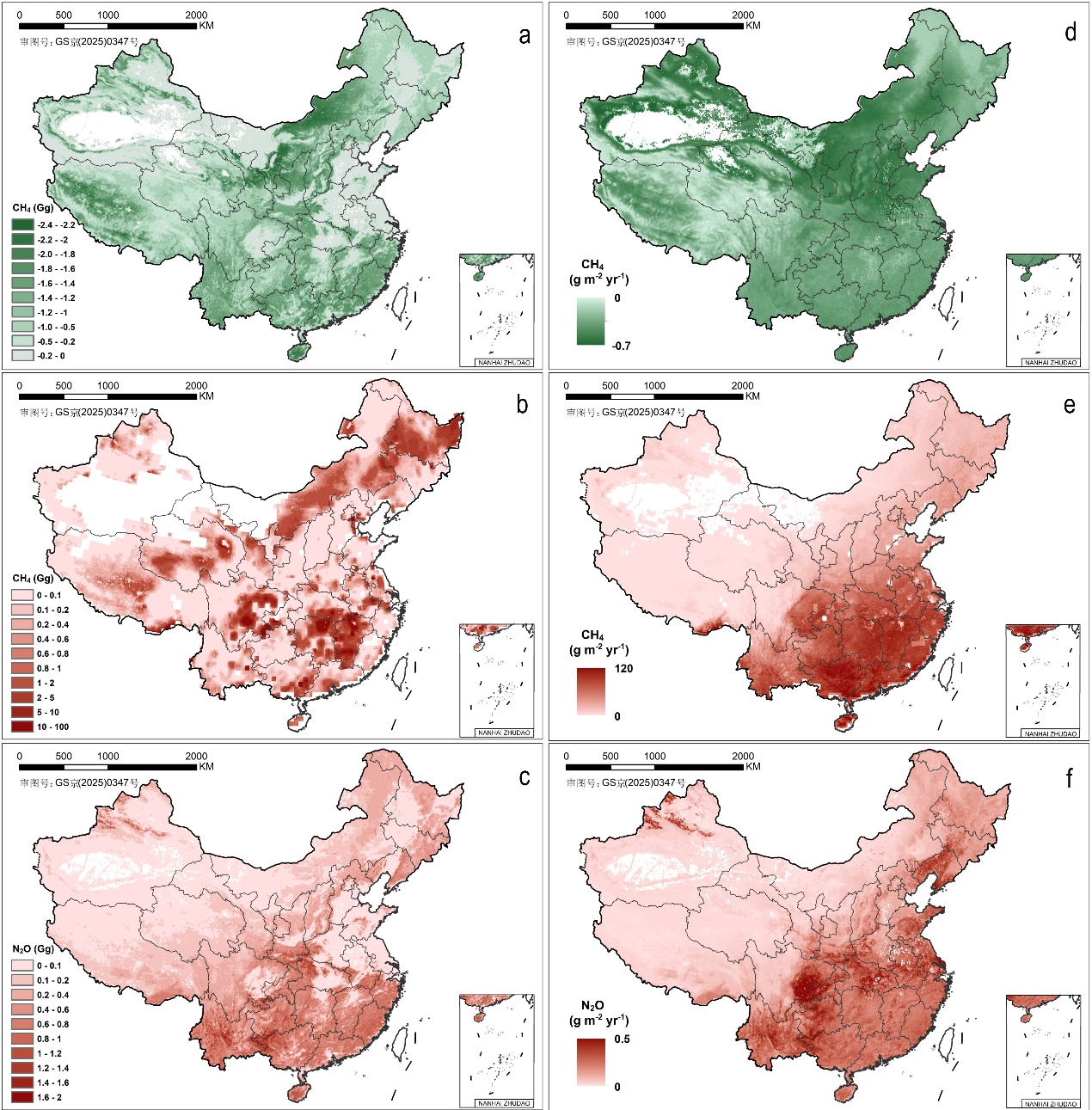
**

Fig. S2 Spatial distribution of cumulative CH_4_ uptake (a), wetland CH_4_ emissions (b), and upland N_2_O emissions (c), along with the annual mean flux intensities of upland CH_4_ uptake (d), wetland CH_4_ emissions (e), and upland N_2_O emissions (f) during the period 1980–2020. There is no available data for Taiwan.


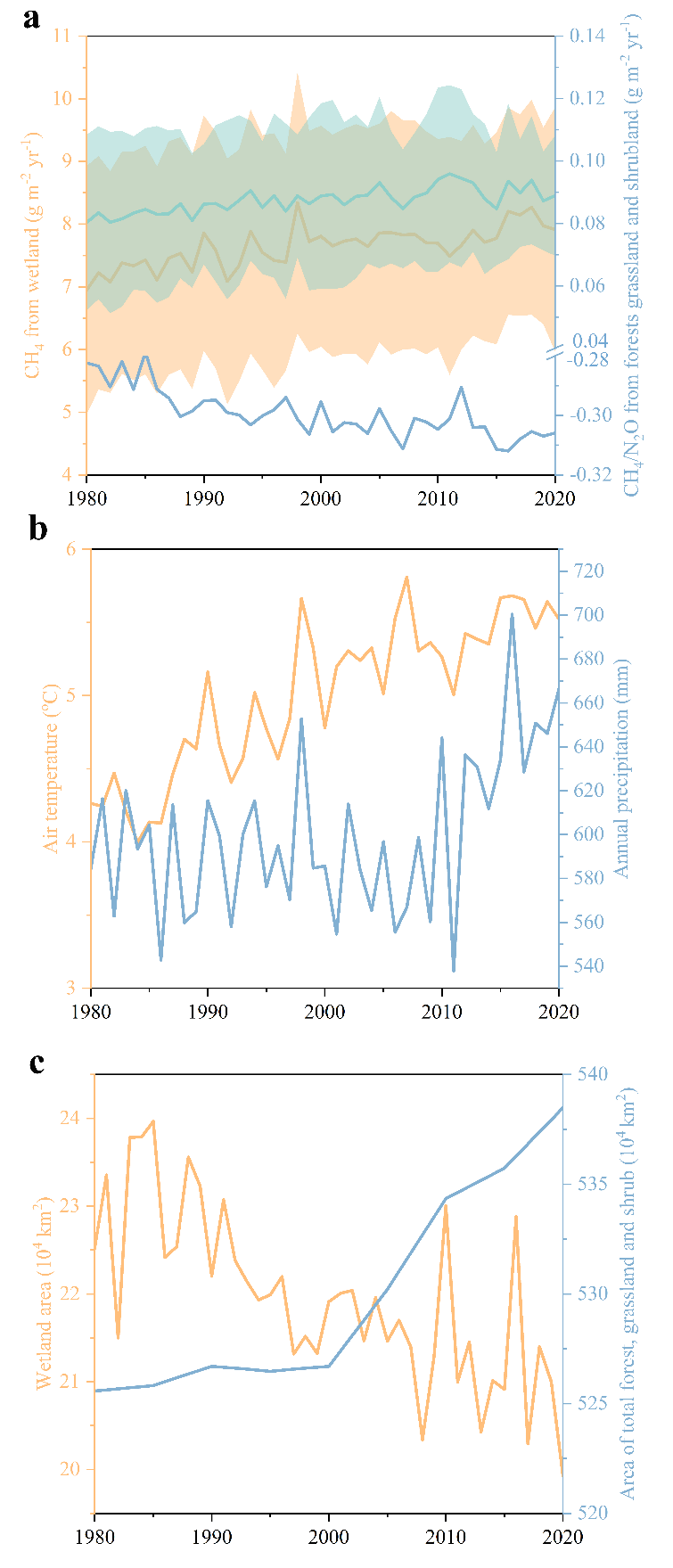


Fig. S3 Temporal variations in flux intensities of (a) wetland CH₄ emissions, upland CH₄ uptake and upland N₂O emissions; (b) air temperature and precipitation and (c), the area of NTEs.

**
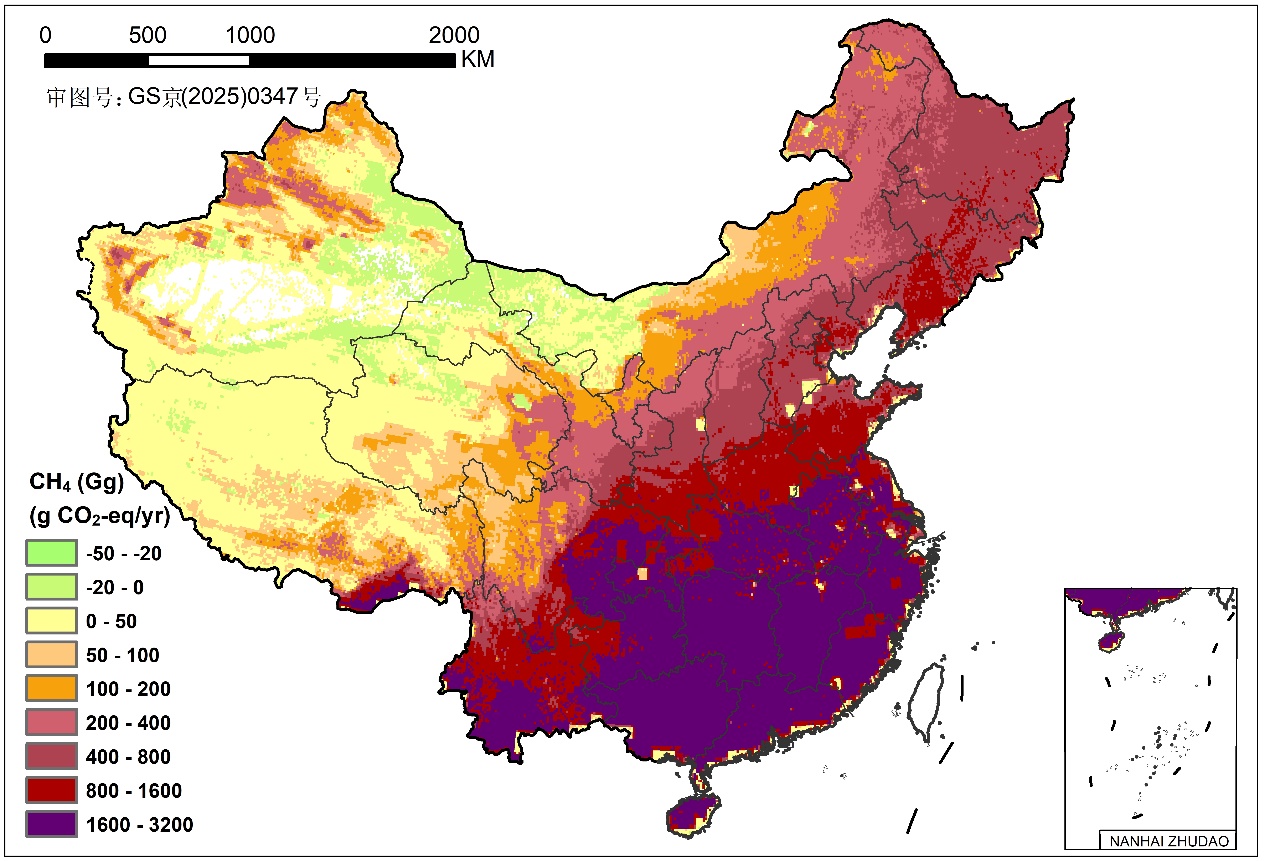
**

Fig. S4 Spatial variations in flux intensities of net GWP from wetland CH_4_ emissions, upland CH_4_ uptake and upland N_2_O emissions. There is no available data for Taiwan.

Reference

1. Xu X, Yuan F, Hanson PJ *et al.* Reviews and syntheses: Four decades of modeling methane cycling in terrestrial ecosystems. *Biogeosciences*. 2016; 13(12): 3735-3755. doi: 10.5194/bg-13-3735-2016

2. Söllinger A, Urich T. Methylotrophic methanogens everywhere—physiology and ecology of novel players in global methane cycling. *Biochem Soc Trans*. 2019; 47(6): 1895-1907. doi: 10.1042/BST20180565

3. Mancinelli RL. The regulation of methane oxidation in soil. *Annu Rev Microbiol*. 1995; 49(1): 581-605. doi: 10.1146/annurev.mi.49.100195.003053

4. Whalen SC. Biogeochemistry of Methane Exchange between Natural Wetlands and the Atmosphere. *Environ Eng Sci*. 2005; 22(1): 73-94. doi: 10.1089/ees.2005.22.73

5. Shukla PN, Pandey KD, Mishra VK. Environmental determinants of soil methane oxidation and methanotrophs. *Crit Rev Environ Sci Technol*. 2013; 43(18): 1945-2011. doi: 10.1080/10643389.2012.672053

6. Leu AO, Cai C, McIlroy SJ *et al.* Anaerobic methane oxidation coupled to manganese reduction by members of the Methanoperedenaceae. *The ISME Journal*. 2020; 14(4): 1030-1041. doi: 10.1038/s41396-020-0590-x

7. Zhao Q, Lu Y. Anaerobic oxidation of methane in terrestrial wetlands: The rate, identity and metabolism. *Sci Total Environ*. 2023; 902: 166049. doi: 10.1016/j.scitotenv.2023.166049

8. Yu L, Huang Y, Zhang W *et al.* Methane uptake in global forest and grassland soils from 1981 to 2010. *Sci Total Environ*. 2017; 607-608: 1163-1172. doi: 10.1016/j.scitotenv.2017.07.082

9. Feng H, Guo J, Han M *et al.* A review of the mechanisms and controlling factors of methane dynamics in forest ecosystems. *Forest Ecol Manag*. 2020; 455: 117702. doi: 10.1016/j.foreco.2019.117702

10. Ding W, Cai Z, Tsuruta H *et al.* Effect of standing water depth on methane emissions from freshwater marshes in northeast China. *Atmos Environ*. 2002; 36: 5149-5157. doi: 10.1016/S1352-2310(02)00647-7

11. Li T, Zhang W, Zhang Q *et al.* Impacts of climate and reclamation on temporal variations in CH_4_ emissions from different wetlands in China: from 1950 to 2010. *Biogeosciences*. 2015; 12(23): 6853–6868. doi: 10.5194/bg-12-6853-2015

12. Ge M, Korrensalo A, Laiho R *et al.* Plant-mediated CH_4_ exchange in wetlands: A review of mechanisms and measurement methods with implications for modelling. *Sci Total Environ*. 2023; 914: 169662. doi: 10.1016/j.scitotenv.2023.169662

13. Aryal B, Gurung R, Camargo AF *et al.* Nitrous oxide emission in altered nitrogen cycle and implications for climate change. *Environ Pollut*. 2022; 314: 120272. doi: 10.1016/j.envpol.2022.120272

14. Colliver B, Stephenson T. Production of nitrogen oxide and dinitrogen oxide by autotrophic nitrifiers. *Biotechnol Adv*. 2000; 18(3): 219-232. doi: 10.1016/S0734-9750(00)00035-5

15. Kelso B, Smith RV, Laughlin RJ *et al.* Dissimilatory nitrate reduction in anaerobic sediments leading to river nitrite accumulation. *Appl Environ Microbiol*. 1997; 63(12): 4679-4685. doi: 10.1128/aem.63.12.4679-4685.1997

16. Lenhart K, Behrendt T, Greiner S *et al.* Nitrous oxide effluxes from plants as a potentially important source to the atmosphere. *New Phytol* 2019; 221(3): 1398-1408. doi: 10.1111/nph.15455

17. Butterbach-Bahl K, Baggs EM, Dannenmann M *et al.* Nitrous oxide emissions from soils: how well do we understand the processes and their controls? *Phil Trans R Soc B*. 2013; 368(1621): 20130122. doi: 10.1098/rstb.2013.0122

18. Ansari J, Udawatta RP, Anderson SH. Soil nitrous oxide emission from agroforestry, rowcrop, grassland and forests in North America: a review. *Agrofor Syst*. 2023; 97(8): 1465-1479. doi: 10.1007/s10457-023-00870-y

19. Brooks PD, Schmidt SK, Williams MW. Winter production of CO_2_ and N_2_O from alpine tundra: environmental controls and relationship to inter-system C and N fluxes. *Oecologia*. 1997; 110(3): 403-413. doi: 10.1007/PL00008814

20. Cen X, Li M, Xu L *et al.* Atmospheric N deposition significantly enhanced soil N_2_O emission from eastern China forests. *Global Biogeochem Cy*. 2022; 36(8): e2021GB007289. doi: 10.1029/2021GB007289

21. Takakai F, Desyatkin AR, Lopez CML *et al.* CH_4_ and N_2_O emissions from a forest-alas ecosystem in the permafrost taiga forest region, eastern Siberia, Russia. *J Geophys Res Biogeosci*. 2008; 113: G02002. doi: 10.1029/2007JG000521

22. Peltola O, Vesala T, Gao Y *et al.* Monthly gridded data product of northern wetland methane emissions based on upscaling eddy covariance observations. *Earth Syst Sci Data*. 2019; 11(3): 1263-1289. doi: 10.5194/essd-2019-28

23. McNicol G, Fluet‐Chouinard E, Ouyang Z *et al.* Upscaling wetland methane emissions from the FLUXNET‐CH4 eddy covariance network (UpCH4 v1. 0): Model development, network assessment, and budget comparison. *AGU Adv*. 2023; 4(5): e2023AV000956. doi: 10.1029/2023AV000956

24. Liao J, Huang Y, Li Z *et al.* Data-driven modeling on the global annual soil nitrous oxide emissions: Spatial pattern and attributes. *Sci Total Environ*. 2023; 903: 166472. doi: 10.1016/j.scitotenv.2023.166472

25. Lang PM, Steele LP, Martin RC *et al.* *Atmospheric methane data for the period 1983–1985 from the NOAA/GMCC global cooperative flask sampling network*: Technical memo, Colorado Univ., Boulder, CO (USA). Cooperative Inst. for Research in Environmental Sciences1990, 1990.

26. Jacob DJ, Varon DJ, Cusworth DH *et al.* Quantifying methane emissions from the global scale down to point sources using satellite observations of atmospheric methane. *Atmos Chem Phys*. 2022; 22(14): 9617-9646. doi: 10.5194/acp-22-9617-2022

27. Wenping Yuan, Minqi Liang, Yuanyi Gao *et al.* China’s greenhouse gas budget during 2000-2023. *Natl Sci Rev*. 2024.

28. Li T, Huang Y, Zhang W *et al.* CH4MOD_wetland_: A biogeophysical model for simulating methane emissions from natural wetlands. *Ecol Model*. 2010; 221(4): 666–680. doi: doi:10.1016/j.ecolmodel.2009.05.017

29. Li T, Lu Y, Yu L *et al.* Evaluation of CH4MOD_wetland_ and Terrestrial Ecosystem Model (TEM) used to estimate global CH_4_ emissions from natural wetlands. *Geosci Model Dev*. 2020; 13(8): 3769–3788. doi: doi:10.5194/gmd-13-3769-2020

30. Zhu Q, Liu J, Peng C *et al.* Modelling methane emissions from natural wetlands by development and application of the TRIPLEX-GHG model. *Geoscientific Model Development*. 2014; 7(3): 981-999.

31. Song C, Luan J, Xu X *et al.* A microbial functional group-based CH4 model integrated into a terrestrial ecosystem model: model structure, site-level evaluation, and sensitivity analysis. *J Adv Model Earth Syst*. 2020; 12(4): e2019MS001867. doi: 10.1029/2019ms001867

32. Murguia-Flores F, Arndt S, Ganesan AL *et al.* Soil Methanotrophy Model (MeMo v1. 0): a process-based model to quantify global uptake of atmospheric methane by soil. *Geosci Model Dev*. 2018; 11(6): 2009-2032. doi: 10.5194/gmd-11-2009-2018

33. Hong S, Li Z, Tang M. Magnitude, distribution and temporal trend of nitrous oxide emissions from China’s natural soils over 1980-2022. *Under review*.

34. Liang M, Zhou Z, Ren P *et al.* Four decades of full-scale nitrous oxide emission inventory in China. *Natl Sci Rev*. 2024; 11: nwad285. doi: 10.1093/nsr/nwad285

35. Ma M, Song C, Fang H *et al.* Development of a Process-Based N2O Emission Model for Natural Forest and Grassland Ecosystems. *J Adv Model Earth Syst*. 2022; 14(3): e2021MS002460. doi: 10.1029/2021MS002460

36. Li T, Zhang Q, Cheng Z *et al.* Performance of CH4MOD wetland for the case study of different regions of natural Chinese wetland. *Journal of Environmental Sciences*. 2017; 57: 356-369.

37. Bohn T, Lettenmaier D, Sathulur K *et al.* Methane emissions from western Siberian wetlands: heterogeneity and sensitivity to climate change. *Environ Res Lett*. 2007; 2(4): 045015. doi: doi:10.1088/1748-9326/2/4/045015

38. Zhang K, Peng C, Wang M *et al.* Process-based TRIPLEX-GHG model for simulating N_2_O emissions from global forests and grasslands: Model development and evaluation. *J Adv Model Earth Syst*. 2017; 9(5): 2079-2102. doi: 10.1002/2017MS000934

39. Zhu Q, Peng C, Liu J *et al.* Climate-driven increase of natural wetland methane emissions offset by human-induced wetland reduction in China over the past three decades. *Sci Rep*. 2016; 6: 38020. doi: 10.1038/srep38020

40. Zhu Q, Peng CH, Chen H *et al.* Estimating global natural wetland methane emissions using process modelling: spatio-temporal patterns and contributions to atmospheric methane fluctuations. *Global Ecol Biogeogr*. 2015; 24(8): 959–972. doi: doi:10.1111/geb.12307

41. Forster P, Storelvmo T, Armour K *et al.* The earth’s energy budget, climate feedbacks, and climate sensitivity. In: Robert C, H. Damon M, Venkatachalam R (eds.). *Climate Change 2021: The Physical Science Basis Contribution of Working Group I to the Sixth Assessment Report of the Intergovernmental Panel on Climate Change*. Cambridge, United Kingdom and New York, NY, USA: Cambridge University Press; 2021. 923–1054.

42. Arora VK, Melton JR, Plummer D. An assessment of natural methane fluxes simulated by the CLASS-CTEM model. *Biogeosciences*. 2018; 15(15): 4683-4709.

43. Tian H, Xu X, Liu M *et al.* Spatial and temporal patterns of CH 4 and N 2 O fluxes in terrestrial ecosystems of North America during 1979–2008: application of a global biogeochemistry model. *Biogeosciences*. 2010; 7(9): 2673-2694.

44. Riley WJ, Subin ZM, Lawrence DM *et al.* Barriers to predicting changes in global terrestrial methane fluxes: analyses using CLM4Me, a methane biogeochemistry model integrated in CESM. *Biogeosciences*. 2011; 8(7): 1925-1953. doi: 10.5194/bg-8-1925-2011

45. Shu S, Jain AK, Kheshgi HS. Investigating wetland and nonwetland soil methane emissions and sinks across the contiguous United States using a land surface model. *Global Biogeochemical Cycles*. 2020; 34(7): e2019GB006251.

46. Kleinen T, Mikolajewicz U, Brovkin V. Terrestrial methane emissions from the Last Glacial Maximum to the preindustrial period. *Climate of the Past*. 2020; 16(2): 575-595.

47. Gedney N, Huntingford C, Comyn-Platt E *et al.* Significant feedbacks of wetland methane release on climate change and the causes of their uncertainty. *Environmental Research Letters*. 2019; 14(8): 084027. doi: 10.1088/1748-9326/ab2726

48. Wania R, Ross I, Prentice I. Implementation and evaluation of a new methane model within a dynamic global vegetation model: LPJ-WHyMe v1. 3.1. *Geoscientific Model Development*. 2010; 3(2): 565-584.

49. Kleinen T, Brovkin V, Schuldt R. A dynamic model of wetland extent and peat accumulation: results for the Holocene. *Biogeosciences*. 2012; 9(1): 235-248.

50. Zhang Z, Zimmermann NE, Kaplan JO *et al.* Modeling spatiotemporal dynamics of global wetlands: comprehensive evaluation of a new sub-grid TOPMODEL parameterization and uncertainties. *Biogeosciences*. 2016; 13(5): 1387-1408.

51. Stocker B, Spahni R, Joos F. DYPTOP: a cost-efficient TOPMODEL implementation to simulate sub-grid spatio-temporal dynamics of global wetlands and peatlands. *Geoscientific model development (GMD)*. 2014; 7(6): 3089-3110.

52. Spahni R, Wania R, Neef L *et al.* Constraining global methane emissions and uptake by ecosystems. *Biogeosciences*. 2011; 8(6): 1643-1665. doi: 10.5194/bg-8-1643-2011

53. Ringeval B, Friedlingstein P, Koven C *et al.* Climate-CH_4_ feedback from wetlands and its interaction with the climate-CO_2_ feedback. *Biogeosciences*. 2011; 8(8): 2137-2157. doi: 10.5194/bg-8-2137-2011

54. J BD, Woodward FI. Vegetation and the Terrestrial Carbon Cycle: Modelling the First 400 Million Years. Cambridge Univ. Press: JSTOR; 2001.

55. Singarayer JS, Valdes PJ, Friedlingstein P *et al.* Late Holocene methane rise caused by orbitally controlled increase in tropical sources. *Nature*. 2011; 470(7332): 82-85.

56. Zhuang Q, Melillo JM, Kicklighter DW *et al.* Methane fluxes between terrestrial ecosystems and the atmosphere at northern high latitudes during the past century: A retrospective analysis with a process-based biogeochemistry model. *Global Biogeochem Cy*. 2004; 18(3): GB3010. doi: 10.1029/2004gb002239

57. Liu L, Zhuang Q, Oh Y *et al.* Uncertainty quantification of global net methane emissions from terrestrial ecosystems using a mechanistically based biogeochemistry model. *Journal of Geophysical Research: Biogeosciences*. 2020; 125(6): e2019JG005428.

58. Ito A, Inatomi M. Use of a process-based model for assessing the methane budgets of global terrestrial ecosystems and evaluation of uncertainty. *Biogeosciences*. 2012; 9(2): 759–773. doi: doi: 10.5194/bg-9-759-2012

59. Potter CS, Davidson EA, Verchot LV. Estimation of global biogeochemical controls and seasonality in soil methane consumption. *Chemosphere*. 1996; 32(11): 2219-2246. doi: 10.1016/0045-6535(96)00119-1

60. Ridgwell AJ, Marshall SJ, Gregson K. Consumption of atmospheric methane by soils: A process-based model. *Global Biogeochem Cy*. 1999; 13(1): 59-70. doi: 10.1029/1998gb900004

61. Curry C. Modeling the soil consumption of atmospheric methane at the global scale. *Global Biogeochem Cy*. 2007; 21(4): GB4012. doi: 10.1029/2006GB002818

62. Ito A, Patra PK, Umezawa T. Bottom‐Up Evaluation of the Methane Budget in Asia and Its Subregions. *Global Biogeochem Cy*. 2023; 37(6): e2023GB007723. doi: 10.1029/2023GB007723

63. Zhuang Q, Chen M, Xu K *et al.* Response of global soil consumption of atmospheric methane to changes in atmospheric climate and nitrogen deposition. 2013; 27(3): 650-663.

64. Cen X, Li M, Xu L *et al.* Atmospheric N deposition significantly enhanced soil N2O emission from eastern China forests. *Global Biogeochemical Cycles*. 2022; 36(8): e2021GB007289.

65. Hansen S, Jensen H, Nielsen N *et al.* Simulation of nitrogen dynamics and biomass production in winter wheat using the Danish simulation model DAISY. *Fertilizer research*. 1991; 27: 245-259.

66. Nielsen S, Anastácio P, Frias A *et al.* CRISP-crayfish rice integrated system of production. 5. Simulation of nitrogen dynamics. *Ecol Model*. 1999; 123(1): 41-52. doi: 10.1016/S0304-3800(99)00166-0

67. Potter CS, Matson PA, Vitousek PM *et al.* Process modeling of controls on nitrogen trace gas emissions from soils worldwide. *Journal of Geophysical Research: Atmospheres*. 1996; 101(D1): 1361-1377.

68. Parton WJ, Hartman M, Ojima D *et al.* DAYCENT and its land surface submodel: description and testing. *Global Planetary Change*. 1998; 19(1-4): 35-48.

69. Li C, Aber J, Stange F *et al.* A process‐oriented model of N_2_O and NO emissions from forest soils: 1. Model development. *J Geophys Res Atmos*. 2000; 105(D4): 4369-4384. doi: 10.1029/1999JD900949

70. Li C, Cui J, Sun G *et al.* Modeling impacts of management on carbon sequestration and trace gas emissions in forested wetland ecosystems. *Environmental management*. 2004; 33(1): S176-S186.

71. Tsuruta A, Aalto T, Backman L *et al.* Global methane emission estimates for 2000–2012 from CarbonTracker Europe-CH4 v1.0. *Geosci Model Dev*. 2017; 10(3): 1261-1289. doi: 10.5194/gmd-10-1261-2017

72. Ishizawa M, Mabuchi K, Shirai T *et al.* Inter-annual variability of summertime CO2 exchange in Northern Eurasia inferred from GOSAT XCO2. *Environmental Research Letters*. 2016; 11(10): 105001.

73. Zheng B, Chevallier F, Ciais P *et al.* Rapid decline in carbon monoxide emissions and export from East Asia between years 2005 and 2016. *Environmental Research Letters*. 2018; 13(4): 044007.

74. Patra PK, Takigawa M, Watanabe S *et al.* Improved chemical tracer simulation by MIROC4. 0-based atmospheric chemistry-transport model (MIROC4-ACTM). *Sola*. 2018; 14: 91-96.

75. Niwa Y, Tomita H, Satoh M *et al.* A 4D-Var inversion system based on the icosahedral grid model (NICAM-TM 4D-Var v1. 0)–Part 1: Offline forward and adjoint transport models. *Geoscientific Model Development*. 2017; 10(3): 1157-1174.

76. Niwa Y, Fujii Y, Sawa Y *et al.* A 4D-Var inversion system based on the icosahedral grid model (NICAM-TM 4D-Var v1. 0)–Part 2: Optimization scheme and identical twin experiment of atmospheric CO 2 inversion. *Geoscientific Model Development*. 2017; 10(6): 2201-2219.

77. Kaiser W, Zhuravlev R, Ganshin A *et al.* Technical note: A high-resolution inverse modelling technique for estimating surface CO2 fluxes based on the NIES-TM-FLEXPART coupled transport model and its adjoint. *Atmospheric Chemistry and Physics*. 2021; 21(2): 1245–1266.

78. Segers A, Houweling S. Description of the CH4 inversion production chain. *CAMS (Copernicus Atmospheric Monitoring Service) Report*. 2020: latest version: <https://atmosphere>. copernicus. eu/sites/default/files/2018-2011/CAMS2073_2015SC2013_D2073, 2012.

79. McNorton J, Wilson C, Gloor M *et al.* Attribution of recent increases in atmospheric methane through 3-D inverse modelling. *Atmospheric Chemistry Physics*. 2018; 18(24): 18149-18168.

80. Zhao M, Tian X, Wang Y *et al.* Slowdown in China's methane emission growth. *Natl Sci Rev*. 2024; 11(8): nwae223. doi: 10.1093/nsr/nwae223

81. Khalil M, Shearer M, Rasmussen R. Methane sources in China: historical and current emissions. *Chemosphere*. 1993; 26(1): 127-142. doi: 10.1016/0045-6535(93)90417-4

82. Xu X, Tian H. Methane exchange between marshland and the atmosphere over China during 1949–2008. *Global Biogeochem Cy*. 2012; 26(2): GB2006. doi: 10.1029/2010GB003946

83. Li T, Zhang W, Zhang Q *et al.* Impacts of climate and reclamation on temporal variations in CH4 emissions from different wetlands in China: from 1950 to 2010. *Biogeosciences*. 2015; 12(23): 6853-6868.

84. Wei D, Wang X. CH4 exchanges of the natural ecosystems in China during the past three decades: The role of wetland extent and its dynamics. *Journal of Geophysical Research: Biogeosciences*. 2016; 121(9): 2445-2463.

85. Wang M, Dai A, Huang J *et al.* Sources of methane in China: rice fields, agricultural waste treatment, cattle, coal mines, and other minor sources. *Scientia Atmospherica Sinica*. 1993; 17(1): 52-64.

86. Jin H, Wu J, Cheng G *et al.* Methane emissions from wetlands on the Qinghai-Tibet Plateau. *Chinese Science Bulletin*. 1999; 44: 2282-2286.

87. Wang XK, Lu F, Yang L. Methane emissions from China’s natural wetlands: measurements, temporal variations and influencing factors. *Recarbonization of the Biosphere: Ecosystems and the Global Carbon Cycle*. 2012: 99-125.

88. Cai Z. Greenhouse gas budget for terrestrial ecosystems in China. *Science China Earth Sciences*. 2012; 55: 173-182.

89. Ding W, Cai Z. Methane emission from natural wetlands in China: summary of years 1995–2004 studies. *Pedosphere*. 2007; 17(4): 475-486.

90. Chen H, Zhu Qa, Peng C *et al.* Methane emissions from rice paddies natural wetlands, lakes in China: synthesis new estimate. *Global Change Biol*. 2013; 19(1): 19-32. doi: 10.1111/gcb.12034

91. Saunois M, Martinez A, Poulter B *et al.* Global Methane Budget 2000–2020. *Earth Syst Sci Data Discuss*. 2024; 2024: 1–147. doi: 10.5194/essd-2024-115

92. Zhang X, Jiang H, Lu X *et al.* Estimate of methane release from temperate natural wetlands using ENVISAT/SCIAMACHY data in China. *Atmos Environ*. 2013; 69: 191-197. doi: 10.1016/j.atmosenv.2012.12.023

93. NGHGI. The People's Republic of China Thrid National Communications on Climate Change (in Chinese). [*https://unfcccint/sites/default/files/resource/China_NC3_Chinese_0pdf*](https://unfcccint/sites/default/files/resource/China_NC3_Chinese_0pdf). 2018.

94. NGHGI. The People's Republic of China Forth National Communications on Climate Change (in Chinese). [*https://unfcccint/sites/default/files/resource/China_NC4_Chinesepdf*](https://unfcccint/sites/default/files/resource/China_NC4_Chinesepdf). 2023.

95. Xiao D, Deng L, Kim DG *et al.* Carbon budgets of wetland ecosystems in China. *Global Change Biology*. 2019; 25(6): 2061-2076.

96. Tian H, Xu X, Lu C *et al.* Net exchanges of CO2, CH4, and N2O between China's terrestrial ecosystems and the atmosphere and their contributions to global climate warming. *Journal of Geophysical Research: Biogeosciences*. 2011; 116(G2).

97. Wang Y, Chen H, Zhu Q *et al.* Soil methane uptake by grasslands and forests in China. *Soil Biology and Biochemistry*. 2014; 74: 70-81.

98. Tian H, Yang J, Xu R *et al.* Global soil nitrous oxide emissions since the preindustrial era estimated by an ensemble of terrestrial biosphere models: Magnitude, attribution, and uncertainty. *Global Change Biol*. 2019; 25(2): 640–659. doi: 10.1111/gcb.14514

99. Luo Y. Estimation of nitrous oxide emissions from forests and grasslands in China. Northwest Agriculture and Forestry Technology University, 2015.

100. Chen G, Huang B, Xu H *et al.* Nitrous oxide emissions from terrestrial ecosystems in China. *Chemosphere-Global Change Science*. 2000; 2(3-4): 373-378.

101. Xu-Ri, Wang Y, Wang Y *et al.* Estimating N2O emissions from soils under natural vegetation in China. *Plant and Soil*. 2019; 434: 271-287.

102. Li S, Bush RT, Santos IR *et al.* Large greenhouse gases emissions from China's lakes and reservoirs. *Water Res*. 2018; 147: 13−24. doi: 10.1016/j.watres.2018.09.053

103. Muñoz-Sabater J, Dutra E, Agustí-Panareda A *et al.* ERA5-Land: A state-of-the-art global reanalysis dataset for land applications. *Earth Sys Sci Data*. 2021; 13(9): 4349-4383.

104. SubCenter S. National Earth System Science Data Center, National Science & Technology Infrastructure of China (<http://soil.geodata.cn>).

105. Li T, Canadell JG, Yang X-Q *et al.* Methane emissions from wetlands in China and their climate feedbacks in the 21st century. *Environmental Science Technology*

2022; 56(17): 12024-12035.

106. Zhang Z, Fluet-Chouinard E, Jensen K *et al.* Development of the global dataset of Wetland Area and Dynamics for Methane Modeling (WAD2M). *Earth Syst Sci Data*. 2021; 13(5): 2001–2023. doi: 10.5194/essd-13-2001-2021

107. Fluet-Chouinard E, Stocker BD, Zhang Z *et al.* Extensive global wetland loss over the past three centuries. *Nature*. 2023; 614(7947): 281–286. doi: doi:10.1038/s41586-022-05572-6

108. Xia X, Xia J, Chen X *et al.* Reconstructing Long‐Term Forest Cover in China by Fusing National Forest Inventory and 20 Land Use and Land Cover Data Sets. *J Geophys Res Biogeosci*. 2023; 128(4): e2022JG007101. doi: 10.1029/2022JG007101
